# Mussel‐inspired biomaterials: From chemistry to clinic

**DOI:** 10.1002/btm2.10385

**Published:** 2022-08-11

**Authors:** Ali Taghizadeh, Mohsen Taghizadeh, Mohsen Khodadadi Yazdi, Payam Zarrintaj, Joshua D. Ramsey, Farzad Seidi, Florian J. Stadler, Haeshin Lee, Mohammad Reza Saeb, Masoud Mozafari

**Affiliations:** ^1^ Institute of Tissue Regeneration Engineering (ITREN), Dankook University Cheonan Republic of Korea; ^2^ Center of Excellence in Electrochemistry School of Chemistry, College of Science, University of Tehran Tehran Iran; ^3^ School of Chemical Engineering, Oklahoma State University Stillwater Oklahoma USA; ^4^ Jiangsu Co‐Innovation Center of Efficient Processing and Utilization of Forest Resources and International Innovation Center for Forest Chemicals and Materials Nanjing Forestry University Nanjing China; ^5^ College of Materials Science and Engineering Shenzhen Key Laboratory of Polymer Science and Technology Guangdong China; ^6^ Department of Chemistry Korea Advanced Institute of Science and Technology (KAIST) Daejeon Republic of Korea; ^7^ Department of Polymer Technology, Faculty of Chemistry Gdańsk University of Technology Gdańsk Poland; ^8^ Department of Tissue Engineering & Regenerative Medicine Iran University of Medical Sciences Tehran Iran; ^9^ Present address: Lunenfeld‐Tanenbaum Research Institute Mount Sinai Hospital, University of Toronto Toronto, ON Canada

**Keywords:** biomaterials, biomedical applications, catechol, coacervation, mussel‐inspired chemistry, pyrogallol, wet adhesion

## Abstract

After several billions of years, nature still makes decisions on its own to identify, develop, and direct the most effective material for phenomena/challenges faced. Likewise, and inspired by the nature, we learned how to take steps in developing new technologies and materials innovations. Wet and strong adhesion by *Mytilidae* mussels (among which *Mytilus edulis* —blue mussel and *Mytilus californianus* —California mussel are the most well‐known species) has been an inspiration in developing advanced adhesives for the moist condition. The wet adhesion phenomenon is significant in designing tissue adhesives and surgical sealants. However, a deep understanding of engaged chemical moieties, microenvironmental conditions of secreted proteins, and other contributing mechanisms for outstanding wet adhesion mussels are essential for the optimal design of wet glues. In this review, all aspects of wet adhesion of Mytilidae mussels, as well as different strategies needed for designing and fabricating wet adhesives are discussed from a chemistry point of view. Developed muscle‐inspired chemistry is a versatile technique when designing not only wet adhesive, but also, in several more applications, especially in the bioengineering area. The applications of muscle‐inspired biomaterials in various medical applications are summarized for future developments in the field.

## 1 INTRODUCTION

From antiquity and ancient times, humankind has always been inspiring by the nature to make proficient use and design materias. Natural evolution has spent millions of years balancing and optimizing almost everything. Correspondingly, every aspect of natural phenomena has been inspired by scientists to mimic the nature from chameleons to leaves. Scientists have deeply been mimicking mechanisms underlying natural changes to find an optimum and lasting solution for addressing different issues engaged with the human body. Researchers who work in the medical field have been attempting to use animals and plants to extract biocompatible materials with minimum toxicity and high efficiency, which could be applied for human disease diagnosis and therapy. These materials must have human tissue characteristics such as matched hydrophilicity and porosity for providing a microenvironment for suitable cell proliferation and food/waste/drug exchanges.^1^, ^2^, ^3^

Since almost two‐third of the human body consists of chameleons adhesives, designing and applying biocompatible materials to firmly adhere to wet surfaces and be capable of holding a significant amount of water in their networks, preserving high mechanical properties against burst and purge pressure, and showing the ability to facile functionalization is in the center has become of particular attention.^4^ Firm adhesion to the wet surfaces along with to severe wave conditions by blue mussels has been an inspiration for the development of novel, sophisticated wet adhesives. Since the degree of interaction between wet adhesive and tissue is the most key factor controlling and determining adhesion efficicncy (particularly when repairing tissue is the target), dealing with chemistry of such adhesives is of critical importance in fabrication of bio‐based adhesives. The mussel chemistry sheds light on the mechanisms of adhesion and helps scientists to design better hydrogels and complexes for biomedical applications.^5^, ^6^, ^7^

## 2 MUSSEL INSPIRED CHEMISTRY

*Mytilidae* mussels have inspired scientists to find a proper attachment in wet conditions, which usually is a complicated process. Blue mussels (*Mytilus edulis*) attach to rocks strong enough such that wind and waves are quite often unable to detach them. This wet adhesion phenomenon has attracted much interest, and numerous investigations have been devoted to reveal the mechanisms and chemicals responsible for this natural adhesion process.^8^, ^9^

**FIGURE 1:**
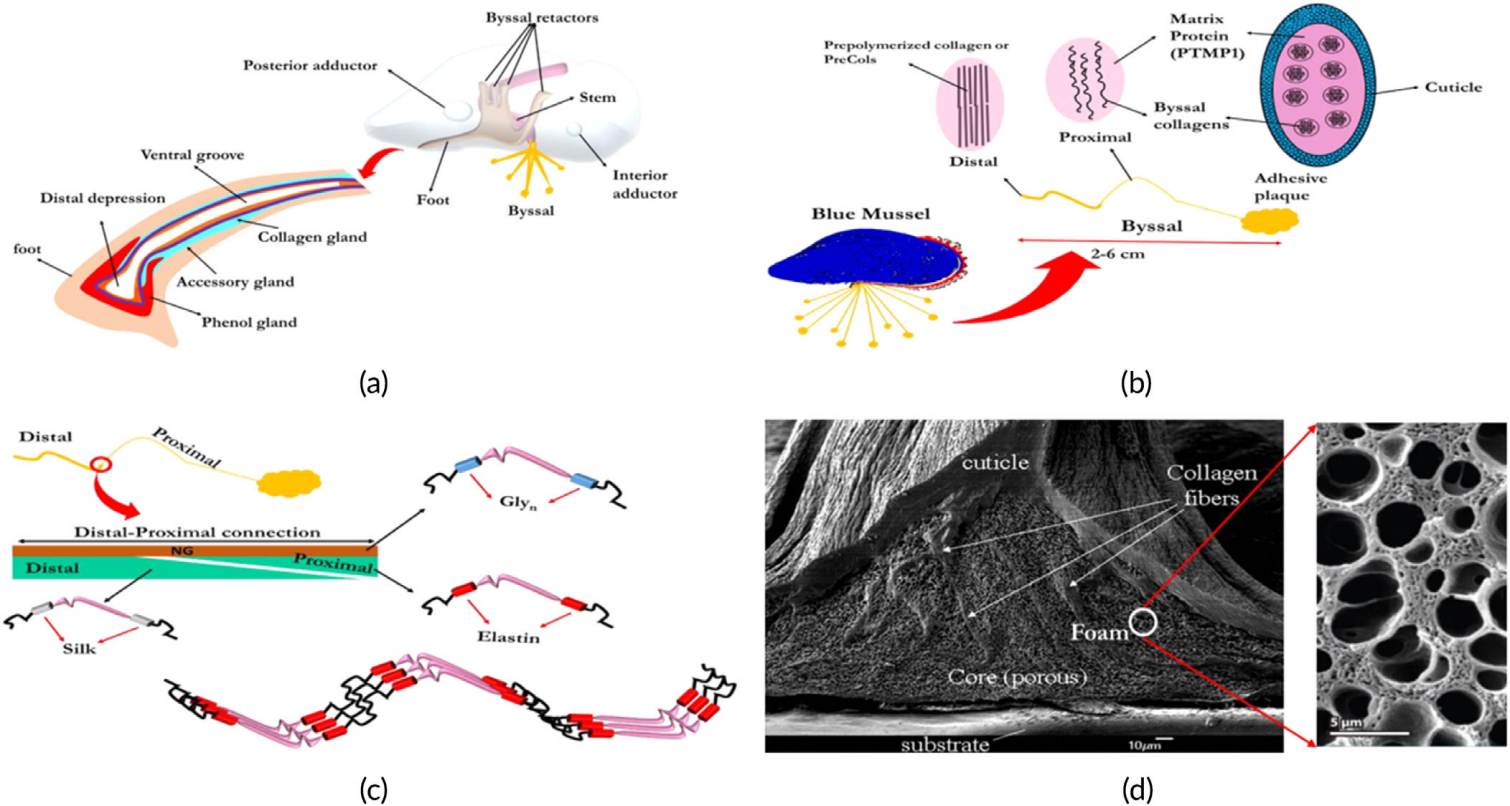
Structure of blue mussel. (a) Different parts and components of the blue mussel and its foot, (b) different parts of the byssal, (c) molecular thread gradients, and (d) SEM image of the plaque adhered to the surface^15^

The mussel's foot is similar to chemical plants that produce constructing materials for manufacturing byssus (i.e., a bundle of hundreds of threads) (Figure 1a). The raw materials are produced within three glands (i.e., phenol, collagen, and accessory glands) in the foot and are delivered through a microfluidic channel known as a ventral groove in which byssal threads are made in a process similar to injection molding of polymers. The foot, like a chemical reactor, provides controlled conditions for the raw materials to react at a defined ratio and makes the core, cuticle, and plaque. In adult mussels, each byssal thread is approximately 2–6 cm in length, composed of three parts of different morphology: (1) adhesive plaque, (2) distal, and (3) proximal sections (Figure 1b).^10^ The elastic proximal section, which is attached to the foot and spread from the stem, is made of loosely packed collagen proteins with a helical structure endowing threads with elasticity and load‐bearing properties.^10^

On the other hand, the distal section is primarily made of tightly packed aligned collagen fibrils (i.e., prepolymerized collagen or PreCols), which provide threads with three kinds of critical flanking regions; silk (truly rigid, 10 GPa), elastin (gentle, 2 GPa), and polyglycine (amorphous and intermediate; Figure 1c).^11^ Such a structure provides a gradual movement from the soft tissue of the foot to the hard substrates (i.e., rocks) at the spatulate plaques.^12^, ^13^ The adhesive plaque, which originates mainly from phenol glands, is the first protein that is injected, followed by the thread core (from collagen glands) and, finally, the cuticle layer (from accessory glands). Cuticle, a 2–5 micron thick layer with granular morphology (Figure 1d), is primarily composed of Mfp‐1, which contains approximately 1 wt% metal ions resulting in high stiffness (2 GPa, compared to underlying collagen fibrils with ~0.5 GPa) and hardness (0.1 GPa). It is worth noting that elongation at break for byssal threads is around 120%.^14^

The adhesive plaque has a porous structure where its voids pattern depends on habitat conditions. This structure is proven to reduce crack propagation and enhance plaque ductility.^16^ However, plaque's chemical structure is the most prominent factor in plaque adhesion. Mussel adhesive proteins (MAPs) secreted from blue mussels, known as mussel foot proteins (Mfp), are responsible for firmly adhering mussels to various surfaces under wet conditions. Six different types of MAP proteins have been identified (Table 1).^17^, ^18^ These proteins are highly cationic (i.e., the MAPs are polyelectrolytes) and have a high content of glycine and 3,4‐dihydroxy‐l‐alanine (DOPA).^19^ Besides, the DOPA has distinguished in secreted materials from *Phragmatopoma californica* (sandcastle worm) and tunicates as well.^10^ DOPA has also been observed in proteins of coral reefs, seashells, and eggshells, where tyrosine oxidation results in DOPA creation.^20^ Other amino acids—namely lysine, phosphoserine, and histidine—appear to contribute to MAP adhesion. DOPA is an amino acid used as a drug for Parkinson's disease. It contains functional OH, COOH, and NH2. DOPA contributes to cohesive and adhesive properties that are essential for any adhesive/glue system. Oxidized DOPA contributes to cohesion while nonoxidized DOPA adheres.^21^

**TABLE 1:** General chemical and physical characteristics of mussel adhesive proteins

	Molecular weight (kDa)	DOPA%	Most abundant proteins	Features	References
Mfp‐1	108	13		Highest weight	22, 23
Mfp‐2	42–47	3		Most abundant in plaques (25 wt%), provide mechanical integrity	22, 23
Mfp‐3‐f	5–7	19%	Glycine (25%), lysine (15%)	DOPA protection against oxidation, diverse variant	23
Mfp‐3‐s		8%	Glycine (29%), tyrosine (19%), Asparagine (18%)		24
Mfp‐4	80	4		Bridging between plaque and byssal thread	22
Mfp‐5	8.9	30.4%	DOPA (30.4%), Lysine (19.8%), Glycine (19.6%)	least polymorphic plaque proteins, hydrophilicity	23
Mfp‐6		3.2%	Tyrosine (19%), Glycine (23.7%), Asparagine (13.4%)		24

**FIGURE 2:**
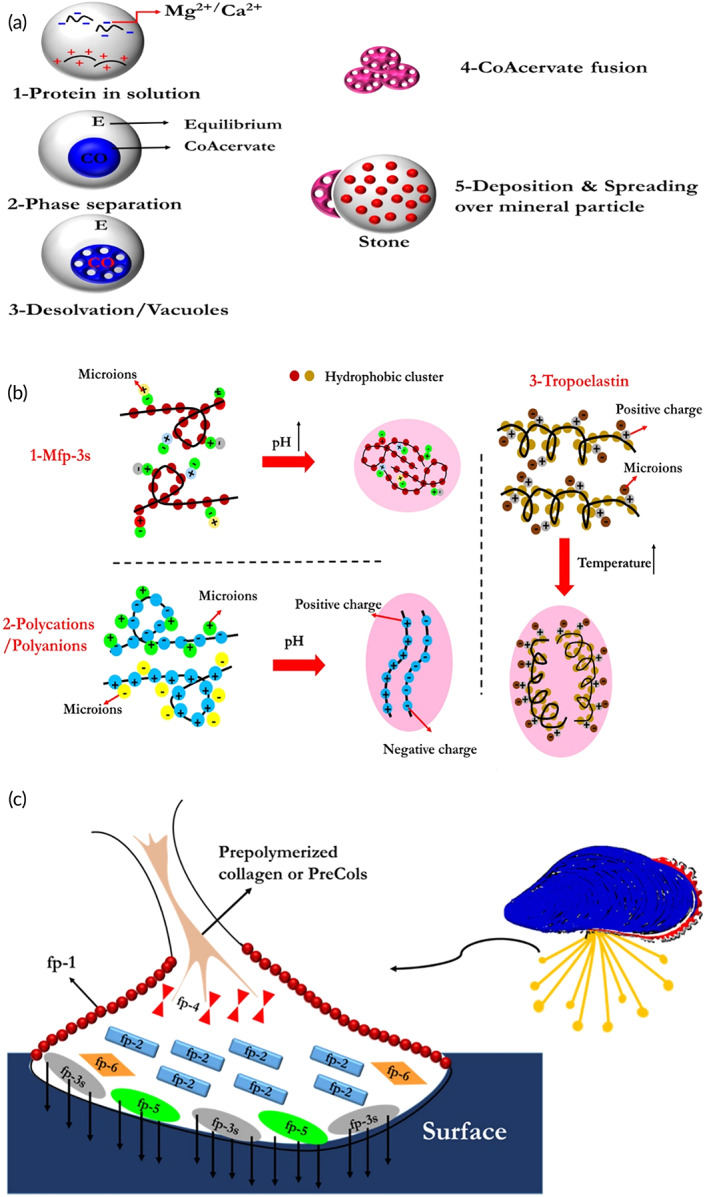
(a) Schematic of various coacervation processes, (b) scheme of adhesive protein coacervation operation, and (c) different types of proteins in byssal plaque^17^

Despite the understanding, the chemical composition of MAPs is fundamental when scrutinizing the mechanism of wet adhesion but realizing the physiochemical conditions of the microenvironment where these MAPs are secreted is also critical. Distal depression of mussel foot resembles inverted cups, which create a cavity of controlled condition for the injection of proteins and further physical and chemical processes. In general, seawater has a high pH value of ~8.1, high ion concentration (~0.7 M), and is saturated with oxygen, while the mussel foot cavities have a lower ion concentration (0.15 M), a lower oxygen concentration, and, thus, a highly reducing and acidic microenvironment (pH = 1–3).^25^, ^26^, ^27^, ^28^ The development of negative gauge pressure in the mussel foot cavity, which triggers suction, enables temporary attachment to the surface, and facilitates protein entry. After these processes, Mfp‐3, Mfp‐5, and Mfp‐6 are secreted and adsorb on the surface while undergoing a liquid–liquid phase separation, known as coacervation, which exhibits the way that cells gather proteins in their fluid‐controlled state. This separated phase will not dissolve easily in aqueous media and shows poor interfacial tension in seawater.^10^ The term coacervation refers to an electrostatically induced liquid–liquid phase separation, for example, when two oppositely charged polyelectrolytes are mixed. All MAPs are positively charged with a high isoelectric point (pI).^29^ However, Mfp‐3S is the unique MAP that can self‐coacervate (Figure 2).^10^, ^17^ Under the physiochemical conditions inside the mussel foot cavity, colloidal suspensions of Mfp‐3S undergo a liquid–liquid phase separation, which results in coacervate formation.^30^ This self‐coacervation process also has a key role in certain biological systems like squid beak formation.^30^ It seems that electrostatic and hydrophobic interactions between nonpolar Mfp‐3S polymers are responsible for phase separation.^29^ Coacervation of MAPs is believed to be the method used by mussels for their initial adhesion to wet surfaces.^29^ The immediate adhesion mechanism of the mold is characterized by a close correlation connecting the coacervate's easy secretion/surface wetting characteristics and the primordial interface sticky function of the Mfp‐5 and Mfp‐3 as surface MAPs, effectively contacting aquatic surfaces.^11^, ^29^ These metastable fluidic coacervates can further undergo phase inversion, crosslinking, and solidification. All in all, the adhesive protein coacervation operation includes several steps. At first, the negative isoelectric point of proteins and positive charges aggregate at acidic region (pH ~ 5); then, the neutralization of opposite charges forms two isolated phases; next, desolvation of equilibrium phase leads to suction and adhesion onto the surface; in the end, secretion and gelation onto the surface take place, thus fixing the byssus thread firmly to the substrate (Figure 2).^11^

Then, the foot is lifted off and seawater, saturated with O_2_, diffuses in, which results in some DOPA (from Mfp‐3 or Mfp‐5) oxidizing to DOPA‐quinone. DOPA‐quinone has poor adhesive but strong cohesive properties due to chemical crosslinking. Mfp‐6 has a low DOPA (3.2%) and a high tyrosine content (19%), while its charged residues (anionic (23%) and cationic (16%) amino acids) are the highest among other types of MAPs.^24^ Further, thiol groups in Mfp‐6, primarily present in cysteine, control the oxidation and reduction reactions DOPA in Mfp‐3 and Mfp‐5.^31^, ^32^ Then, Mfp‐2 and Mfp‐4 are secreted, which form the structure of the adhesive plaque followed by the secretion of other proteins that make threads as discussed earlier. Finally, Mfp‐1 is secreted, which creates the cuticle.

**FIGURE 3:**
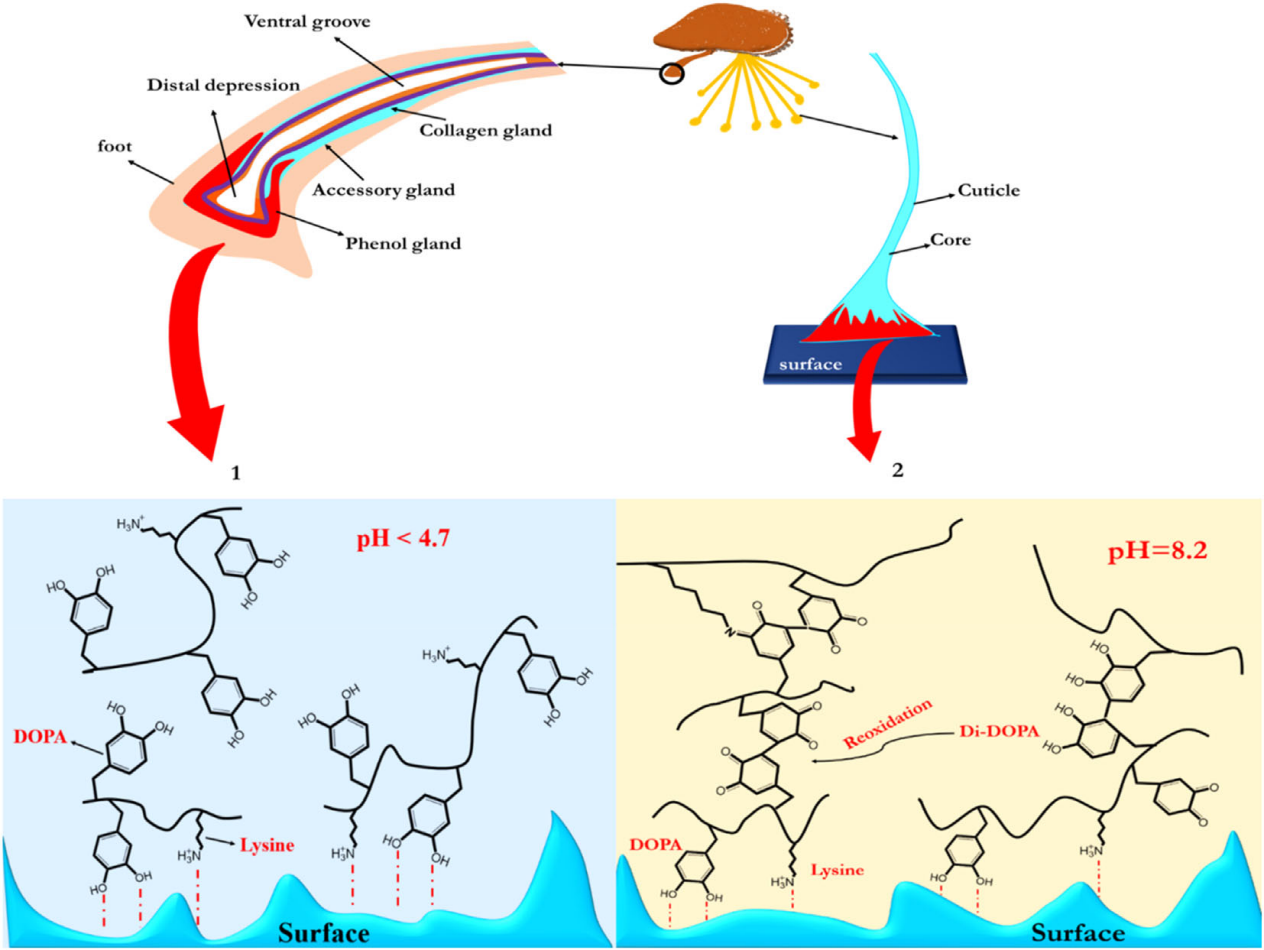
Mussel adhesion to the surface at various pH. (a‐1) at pH < 7, MAPs readily transfer to the surface; Lysine and DOPA facilitate the development of bident H bonds and surface oxide coordination interactions. (a‐2) DOPA's auto‐oxidation at the basic region (pH = 7.5–8.2) is an issue that could cause adhesion to reduce by more than 75% or 95% in comparison to low pH such as 3 and 5, respectively. Along with the formation of dopaquinone, catechol oxidase, and redox transfer between DOPA and iron (III) ions, trigger the formation of crosslinks in the plaque^34^, ^35^

**FIGURE 4:**
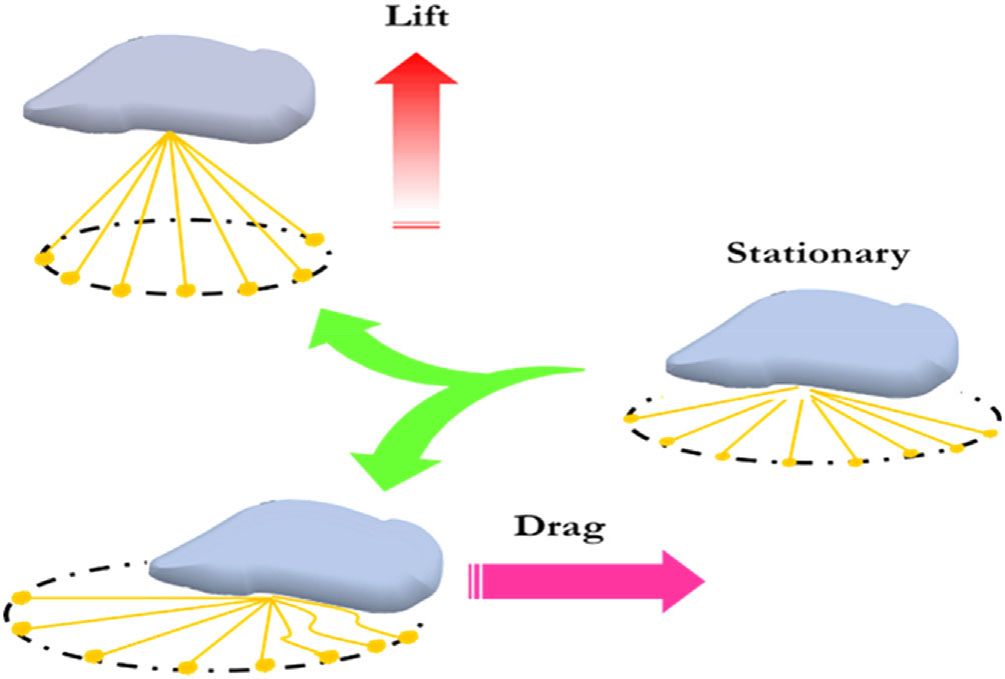
Different positions of threads in various situations

Note that Mfp‐3s, due to possessing higher proportion of hydrophobic amino acid residues compared to Mfp‐3f is capable of inhibiting DOPA from oxidation, especially at pH >7.^33^ It was observed that DOPA moieties have a significantly higher oxidizing ability in Mfp‐3s compared to Mfp‐3f, that is, DOPA is less suspicious of oxidation in the presence of Mfp‐3s, which result in improved adhesion in neutral to basic environments. It is believed that this phenomenon is related to hydrophobic interactions of Mfp‐3s, which results in the creation of a hydrophobic microenvironment encompassing DOPA residues and protecting them from the surrounding environment. The whole process for byssal threads formation takes about 5 min. After 8–12 days of this process, the plaque's adhesive strength increases 100% due to seawater exposure.^34^ This postprocessing phenomenon depends on physiochemical conditions of seawater such as oxygen concentration and pH (Figure 3).^35^ Figure 4 indicates various byssal positions when mussels faced different external forces.

From this perspective, nature using biological processes based on chemical functionality such as dopamine and eugenol inspires scientists to design a material based on mussel chemistry for various uses such as adhesive for biological media. Catechol is the central part of such molecules responsible for adhesiveness. In this regard, we will describe the mussel‐inspired chemistry to pave the way to design appropriate substrates for biomedical applications.

## 3 CATECHOL CONTAINING MATERIALS

There are numerous naturally occurring catechol derivatives with different functionalities, including neurotransmission, pigmentation, surface adhesion, creation of beak and cuticle, crosslinking of proteins, and iron acquisition. For instance, urushiol, catecholamines (such as DOPA, dopamine, norepinephrine, and epinephrine), catechol melanins, catechin, caffeic acid, and tannic acid are some of the natural derivatives of the catechol.^36^, ^37^

**FIGURE 5:**
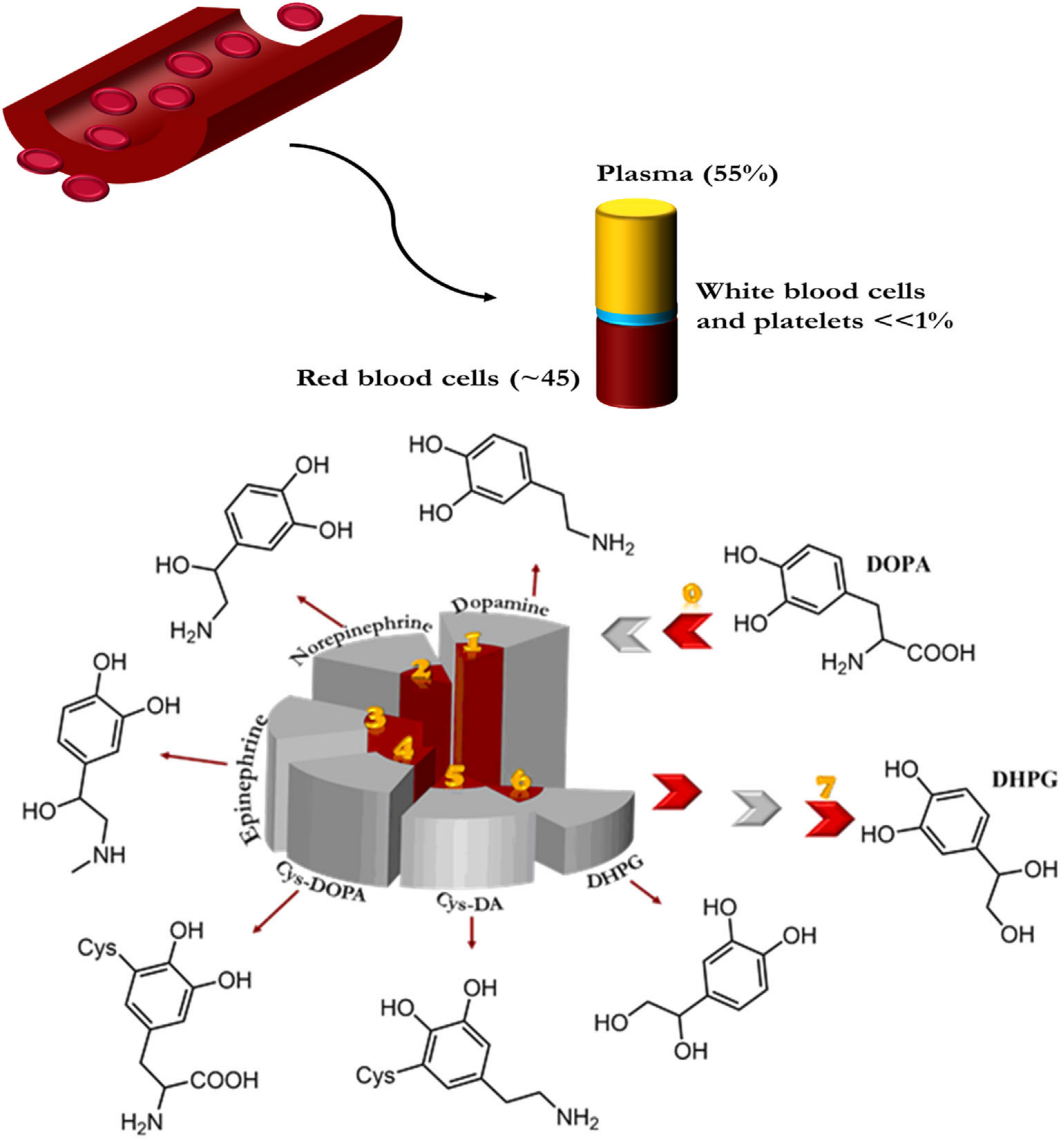
Chemical structures of catechols found in human plasma. Cys 5‐S‐cysteinyl, DOPAC 3,4‐dihydroxy phenylacetic acid, DHPG 3, and 4‐dihydroxy phenyl glycol^43^

Catecholamines are a set of chemical neurotransmitters that contribute to regulating physiological processes while also contributing to several diseases, including cardiovascular, neurological, and endocrine diseases.^38^ Tyrosine and DOPA are the main precursors for manufacturing catecholamines in living organisms.^39^ Dopamine is a neurotransmitter that is very important in the brain and the human blood plasma (Figure 5). Besides, it is a precursor of norepinephrine and epinephrine hormones.^40^ Unbalanced dopamine levels lead to various disorders and illnesses like Parkinson's disease.^41^ Dopamine is found in the central nervous system (CNS), where its axon terminal concentration is maximum for all body parts.^42^

3,4‐Dihydroxy‐catechols are predominantly found in dopamine and its derivatives, such as DOPA, while 2,3‐dihydroxy‐catechols (especially 2,3‐dihydroxybenzoic acid) are found in siderophores secreted by microorganisms, which strongly chelate Fe^3+^ when transporting it through the cell membrane.^44^ Enterobactin is the siderophore of high affinity found primarily in Gram‐negative bacteria such as *Salmonella typhimurium*. The primary function of this siderophore is acquiring iron, as shown in the following figure. Besides animal species, catechol structure can be observed in plant species such as natural dyes like flavonoids (e.g., quercetin), which results in antioxidant and strong radical scavenging potential.^45^, ^46^ Tannic acid, which is a polyphenolic compound, has been recently at the center of attention in biomedical applications.^47^, ^48^ This weak acid rich in pyrogallol/catechol functional groups endow the acid with antioxidant, radical scavenging, and adhesive properties.^49^, ^50^ Caffeic acid is another catechol‐containing carboxylic acid, which is a precursor to the synthesis of lignin in plants.^51^ This natural polyphenol is also found in coffee and certain fruits, oils, and herbs and possesses antioxidant properties.^52^

On the other hand, different catechol‐containing derivatives such as dopamine can be manufactured through substitution on the benzene ring or the side chain.^53^ These substituted functional groups affect the charge distribution on the benzene ring, which alters the reactivity and adhesion properties of catechol groups. Besides, acrylamide derivatives such as N‐(3,4‐dihydroxyphenethyl)methacrylamide have also been used in manufacturing MI polymers.^54^ These derivatives, which have been intensely used in polymer synthesis, would be studied in more detail.

The vicinal hydroxyl groups (catechols) of DOPA are a critical factor in its wet adhesion. The maximum adhesion of Mfp‐3 and Mfp‐5 occurs when pH is less than 3. The adhesion strength significantly diminishes at pH = 5.5 and completely disappears at neutral pH.^25^, ^55^ Thanks to the redox properties of catechol, DOPA serves not only in surface adhesion but also in cohesive forces within the bulk of the adhesive plaque.

## 4 CATECHOL INTERACTIONS

The catechol group consists of two adjacent hydroxyl groups attached to an aromatic ring. There are π‐electrons systems below and above the benzene ring, resulting in a quadrupole load distribution and thus allowing electrostatic interactions. In other words, this electron‐rich system can interact with various species, including cations, anions, neutral metals, and other systems like aromatics. Some of these possible interactions are metal–π interactions, polar molecules–π interactions, aromatic–aromatic interactions (π stacking), donor–acceptor interactions, anion–π interactions, cation–π interactions, C–H–π interactions. It is expected that catechol groups which are composed of two neighboring hydroxyls attached to a benzene ring experience similar interactions.^56^ Besides, OH groups would endow their new interactions with other species. Figure 4. shows different interactions of catechol with other species, some of which are reversible while the others are irreversible, that is, covalent bonds. Chemical interactions between ortho‐quinones and catechol‐based materials are generally grouped into (1) noncovalent and (2) covalent interactions.^57^

**FIGURE 6:**
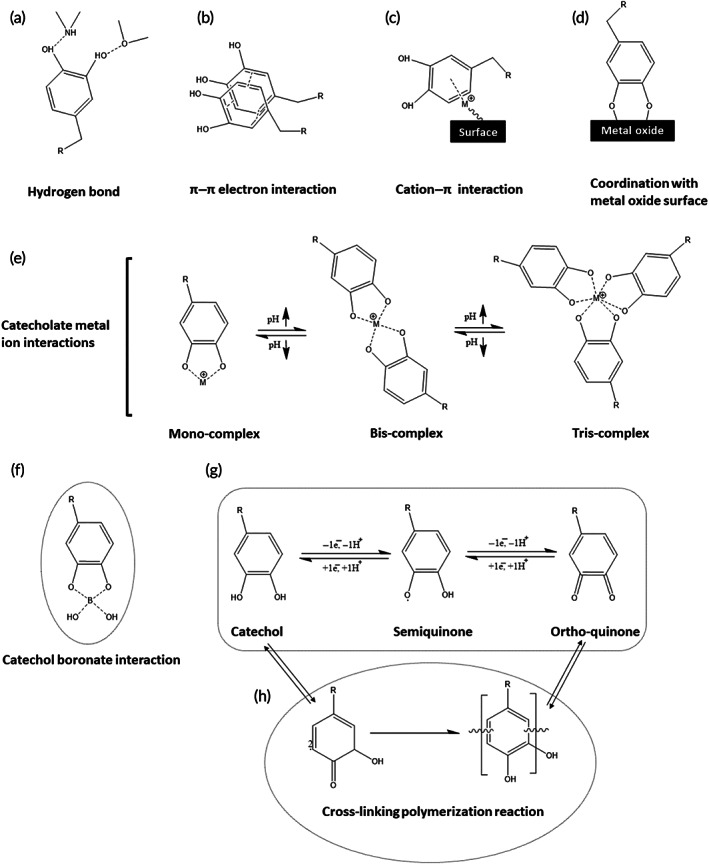
Different interactions between catechol and other moieties

Briefly, the noncovalent interactions related to catechols are subdivided into six classes that are illustrated in Figure 6a–f. The powerful hydrogen bonding between the ortho‐dihydroxy moiety of catechol and polar tails (F, O, N) of surfaces (almost all types of substances, including rocks, hydroxyapatite, and even body tissues) leads to a high adsorbability on surfaces (Figure 6a). Despite the hydrogen bonding, as a polar–polar interaction, catechols, due to the existence of benzene units in their structures (Figure 6b), enable them to build strong π–π interactions with aromatic ring‐containing platforms (e.g., polystyrene).^56^, ^58^ Also, catechols, through an attraction between their benzene rings and cations (cation–π interactions), are capable of adhering to cation‐containing surfaces (Figure 6c).^57^ Figure 6d illustrated that catechols play a role as a robust anchor to modify and functionalize the surface of diverse metal oxides (silver, gold, silicon, and titanium oxides) to nickel–titanium alloys via the reversible interfacial bonds.^59^ Figure 6e explains that the chelation of various metal cations by catechols is suitable to fabricate stable yet reversible complexes, which have many applications in the synthesis of pH‐responsive drug delivery systems, hydrogels with self‐healing features, and soft actuators.^60^, ^61^, ^62^ Also, the reaction between catechols and the boronic acids results in the creation of dynamic covalent bonds between oxygen and boron atoms (Figure 6f).^63^ Catechol can be oxidized in the presence of oxidation agents (e.g., sodium periodate) to produce a radical form of an ortho‐quinone structure (Figure 6g).^57^, ^64^ In addition, catechol oxidase can catalyze the reaction between catechol derivatives and oxygen. In mildly basic aqueous solutions or even in the air, catechols are capable of oxidizing spontaneously. Figure 6h exhibits the fact that dimers were formed whenever quinone molecules start to react with each other or have interactions with other catechols molecules through crosslinking polymerization.^57^

## 5 PHYSICAL/REVERSIBLE INTERACTIONS

Noncovalent interactions (i.e., electrostatic forces, π‐effects, van der Waals [vdW], hydrophobic effect) significantly contribute to biological systems in various ways including fixing protein 3D structure and other biomolecules.^65^, ^66^ These dynamic reversible interactions may also be utilized when designing stimuli‐responsive hydrogels for biomedical applications.^67^ Catechol functional groups contribute to many of these physical interactions summarized in this section.

Catechol benzene interactions with other aromatic species such as polymers (e.g., polystyrene^68^), carbon‐based nanomaterials (e.g., nanotubes and graphenic materials^69^), organic dyes are very common. It was recently revealed that substitution of electron‐donating and removing groups, particularly –OH, on the benzene ring could increase adsorption on graphene sheets.^69^ More interestingly, the adsorption is further enhanced when the number of hydroxyl groups is increased. Adsorption is also weakly related to positions of a disubstituted benzene ring where hydroxyls in para‐position (e.g., hydroquinone) more strongly adhere to graphene compared to ortho‐position (e.g., catechol).^69^

Hydrogen bonds are created between hydrogen atoms, which are bonded to small, highly electronegative atoms (i.e., N‐, O‐, and F‐molecules known as NOF) and adjacent groups with lone pair of electrons (e.g., NH_3_). Strong electrostatic interactions between positively charged hydrogen atoms with negatively charged species carrying single pairs of electrons make H‐bonds stronger than vdW, but weaker than ionic and covalent bonding. Hydrogen bonds can be intermolecular or intramolecular, responsible for exciting phenomena such as the high boiling point of water and secondary and tertiary structures of proteins. In catechol where hydrogen atoms are attached to oxygen atoms, hydrogen bonding can be created. Two hydroxyl groups next to each other can result in various catechol–water clusters in which the strength of hydrogen bonds is different.^70^ Intramolecular hydrogen bonding can endow the catechol‐containing systems with significant antioxidant activity, as well.^71^

Metal complexation crosslinking is common in nature's and nature‐derived bioadhesive, mechanically stressed, and protective coatings with high strength, toughness, self‐healing, wet adhesion, hardness, wear resistance, high extensibility, and adjustable mechanical properties.^72^, ^73^ High hardness, extensibility, and stiffness of the cuticle of byssal mussel threads originate from metal‐l‐DOPA complexes, especially Fe^2+^ and Ca^2+^ ions, which account for approximately 1 wt% of Mfp‐1.^74^ Physical interactions between catechol and metallic ions (i.e., coordination complexes) supply a dynamic physical crosslinking mechanism with high strength.^75^ DOPA‐Fe^3+^ complexation not only contributes to adhesion but also cohesion, but not simultaneously.^76^ Indeed, mussels used Dopa‐Fe^3+^ complexation to shift dopamine functionality from surface adhesion to plaque/substrate interface cohesion. In other words, the complexation of DOPA‐Fe^3+^ leads to the innate surface adhesion that is generated in acidic environments (pH ≈ 2–4) completely fading and strong cohesion between proteins and proteins emerges under basic pH conditions (pH ≈ 8) similar to seawater.

Regardless of the kind of multivalent cations ions, catechol‐containing compounds have a tendency toward adhering to metal and metal oxide surfaces in which the adhesion strength is significantly affected by metal.^15^, ^23^ The metal–catechol bond is one of the strongest physical (or reversible) interactions in biological systems in which 0.8 nN is needed for breaking one single metal–catechol bond compared to covalent bonds such as silicon–carbon (2 nN) and gold–sulfur (1.4 nN).^73^, ^77^ However, the presence of adsorbed water or solvent molecules would affect catechol adsorption on different surfaces.^15^

On the other hand, dihydroxylated compounds with neighboring hydroxyl moieties such as 2‐hydroxy carboxylates, diols, enolizable α‐ketocarboxylates, and catechol derivatives can interact effectively with boronic acids.^78^ Boronate–catechol complexes can prevent DOPA from oxidation.^79^ Thus, these complexes have been used as a temporary protecting group when modifying monomers or polymers with catechol.^80^

## 6 CHEMICAL INTERACTIONS

Metallic cations, especially with high charge density, can interact with the electron‐rich region below/above benzene rings. Electron‐donating groups (e.g., NH_2_, –OH) strengthen this interaction while electron‐withdrawing groups (e.g., CN, NO_2_) weaken it.

### 6.1 Oxidation

**FIGURE 7:**
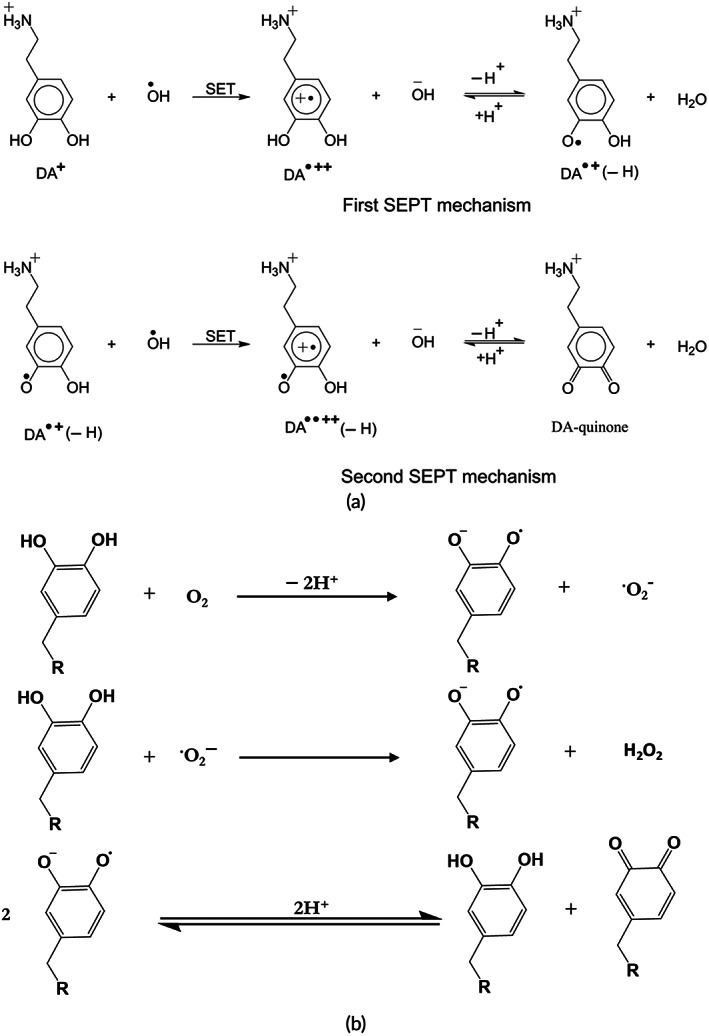
Mechanism of catechol oxidation in (a) water and (b) air^82^

Catechol can be oxidized in the presence of oxidation agents (e.g., sodium periodate, ammonium persulfate, hydrogen peroxide, mushroom tyrosinase, and horseradish peroxidase) into their quinone form. For example, catechol oxidase can catalyze the reaction between catechol derivatives and oxygen. In aqueous, mildly basic solutions, catechol is prone to auto‐oxidation (Figure 7a). Besides, as shown in Figure 7b catechol also spontaneously oxidizes when exposed to air or water.^81^ In all cases, the oxidation is accompanied by a clear “catechol tanning”—the color turns dark red to almost black.^63^

**FIGURE 8:**
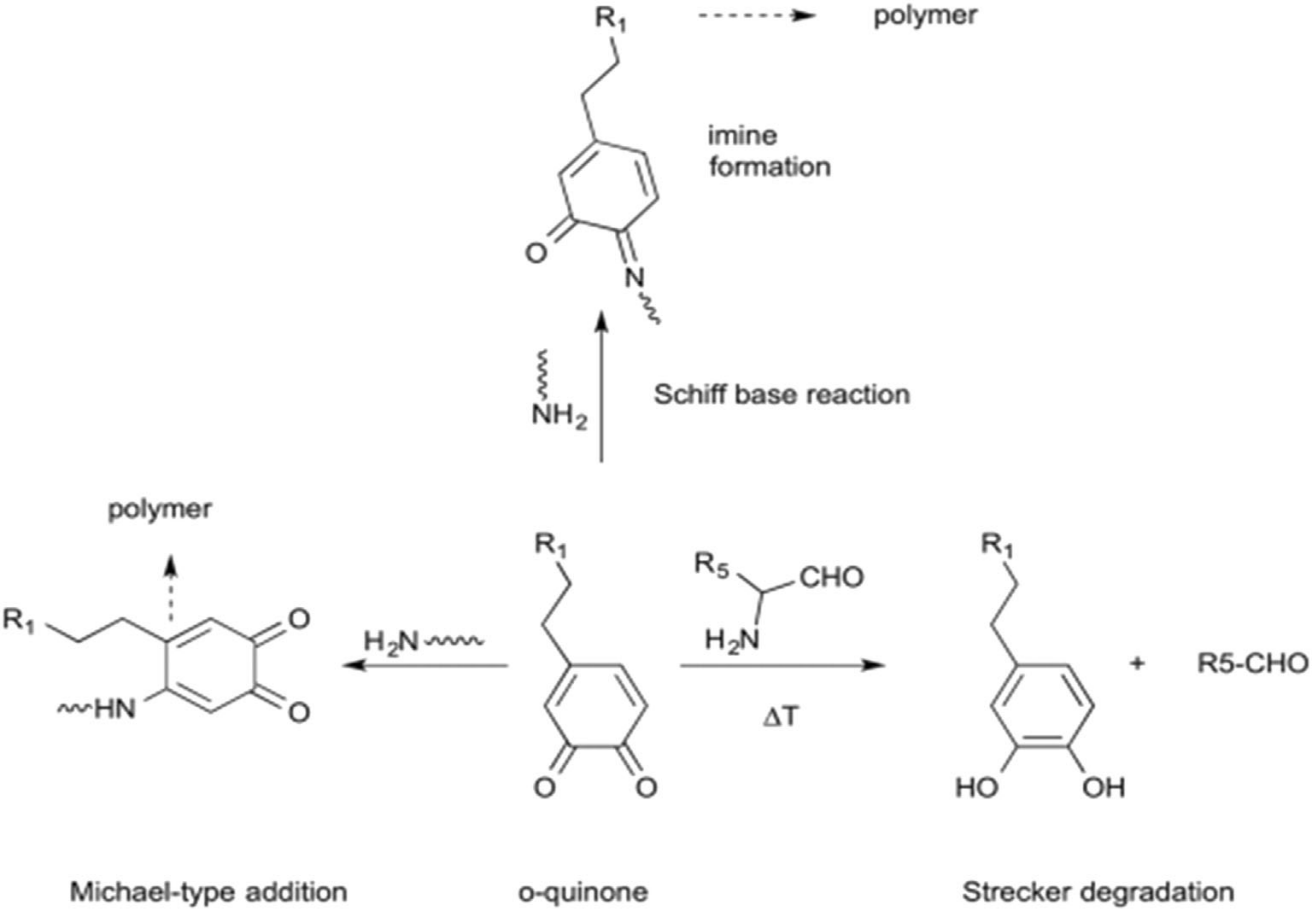
Schematic of Michael addition and Schiff base reaction can occur between quinone and primary amines^84^, ^85^

Quinones are highly reactive intermediates that can be covalently attached to the nucleophilic functional groups (e.g., thiol, thiol acids, primary and secondary amines, cyanamide, or imidazole) via nucleophilic addition reactions (Michael addition or Schiff base substitution as shown in Figure 8).^83^ The reaction of quinones with water yields a 1,4‐addition product which is an unstable intermediate.^39^ Besides, quinones can react with alcohol functional groups through Michael‐1,4‐addition. The reaction between quinones and carboxyl functional groups yields esters.^39^

Furthermore, catecholamine derivatives could be easily oxidized.^39^ Catecholamines (except norepinephrine) oxidize to form semi‐quinone and eventually to quinone form. Quinones reactivity is essential in biological systems. Moreover, a controlled condition in the dopamine microenvironment is essential for adequate brain function.^38^ The oxidation of dopamine may result in Parkinson's disease.^86^ dopamine is a qualified antioxidant that acts as a free radical scavenger protecting neurocytes from oxidative stress.^38^

**FIGURE 9:**
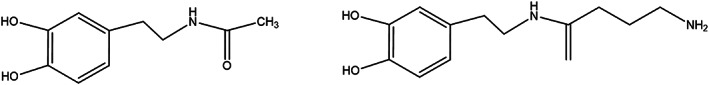
Oxidation of (left) N‐acetyl dopamine and (right) N‐β‐alanyl dopamine

Auto‐oxidation of DOPA at neutral pH is problematic, which can result in more than a 95% decrease in adhesion. DOPA is converted to dopaquinone under oxidizing conditions such as chemical or elevated pH. Nonoxidized DOPA contributes to adhesion, while oxidized DOPA mainly contributes to cohesion.^87^ Oxidation of N‐acetyl dopamine and N‐β‐alanyl dopamine usually occurs during cuticular sclerotization (Figure 9).^88^ The functionalization of the catechol with electron‐withdrawing groups increases the oxidation rate with oxygen, while electron‐donating groups decrease the oxidation rate.

### 6.2 Nucleophilic interactions

**FIGURE 10:**
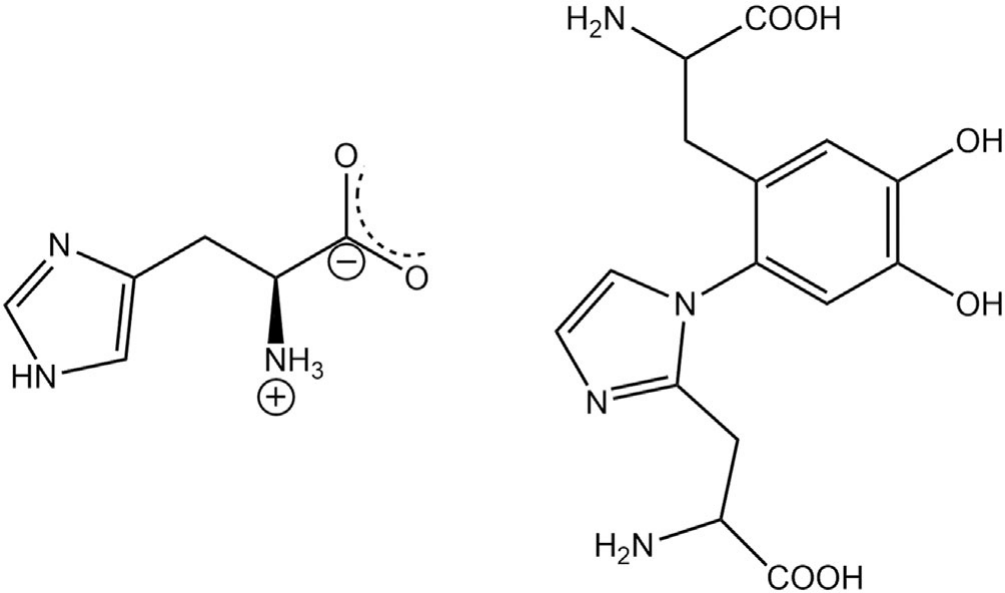
Chemical structure of (left) l‐histidine and (right) DOPA‐histidine^82^

Oxidation intermediates like quinone and quinone methide may react with nucleophilic groups like thiol and amines. The reaction of amine and catechol is critical in many biological systems, including mussel adhesive proteins, polymerization of specific protein subunits to create cytoskeleton in insects, and the synthesis of melanin pigments.^89^ As discussed earlier, along with DOPA, the l‐lysine amino acid is found extensively in blue‐mussel Mfps. The primary amine groups in l‐lysine may undergo reaction with o‐quinone moieties by Schiff‐type reaction, which helps to solidify secreted proteins in blue mussels.^89^ The beak material in jumbo squids is a mineral‐free sclerotized chitinous (the protective outer layer of some species of insects and crustaceans) composite in which covalent crosslinking between DOPA and histidine amino acid is observed in the form of multimers^90^ (Figure 10). Histidine is an essential amino acid that can change into histamine upon decarboxylation. Amine‐catechol chemistry, which is common in biological systems, has focused on References 91 and 92.

The reaction between catechol and thiol is found in many biological systems.^31^ Orthoquinones from the oxidation of catecholamines can react with thiol‐containing cysteine resulting in neurotoxic cysteinyl catecholamine.^93^, ^94^

### 6.3 Oligomerization

**FIGURE 11:**
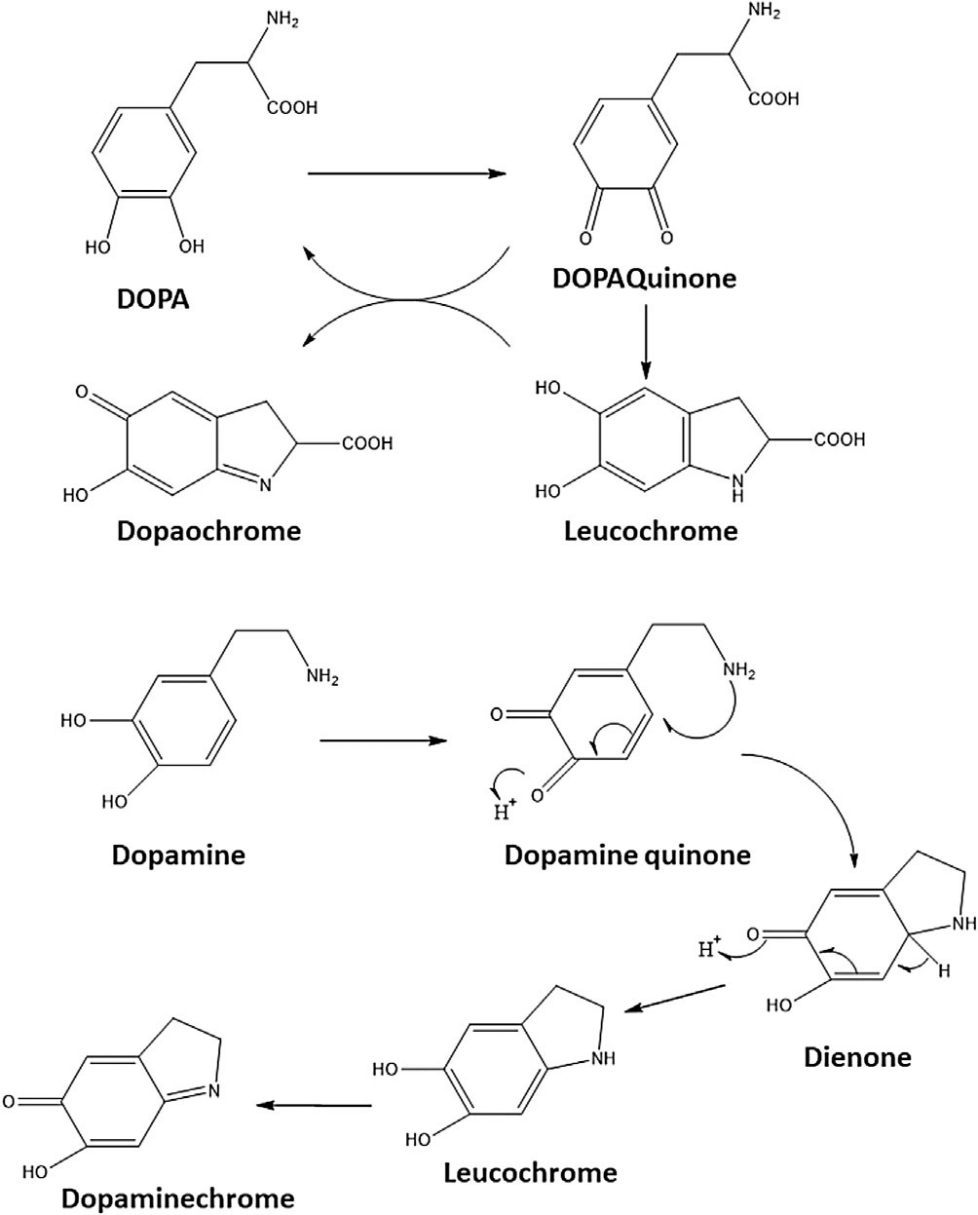
Raper–Mason pathway for the biosynthesis of melanin

The following figure illustrated that DOPA (free amine attached to the catechol group) could undergo intramolecular cyclization after oxidation followed by oligomerization, which results in eumelanin, a group of melanin (Figure 11).^95^

Tyrosinase (A) transforms tyrosine and DOPA into dopaquinone. Dopaquinone undergoes instant intramolecular, nonenzymatic cyclization forming leucochrome, and rapidly oxidizing dopaquinone to dopachrome. Red‐colored dopachrome is transformed as the primary component of 5,6‐dihydroxyindole (DHI) and 5,6‐dihydroxyindole‐2‐carboxylic acid (DHICA). Oxidative dihydroxyindol polymerization produces melanin, the same moiety making human skin darker. Tyrosinase is thought to be the only enzyme associated with this pathway, and the remaining reactions (B) are believed to be nonenzymatic.

### 6.4 Polymerization

Autoxidative dopamine polymerization usually occurs in basic media under oxygen presence.^96^ However, it has recently been reported that dopamine could be polymerized under acidic conditions (pH <5.5).^97^ Plasma‐activated water (PAW), prepared through a micro hollow cathode discharge device, was used as a polymerization medium. The obtained acidic PDA has a similar chemistry to the basic PDA, which is routinely synthesized in basic conditions, but the particles of acidic PDA show superior stability at different pH conditions. This PAW–PDA seems important for large biomedical applications.

DOPA residues can undergo polymerization reactions while forming oligomers with up to six attached monomers, which is responsible for the rapid curing of catechol‐containing adhesives.^92^

### 6.5 Crosslinking

**FIGURE 12:**
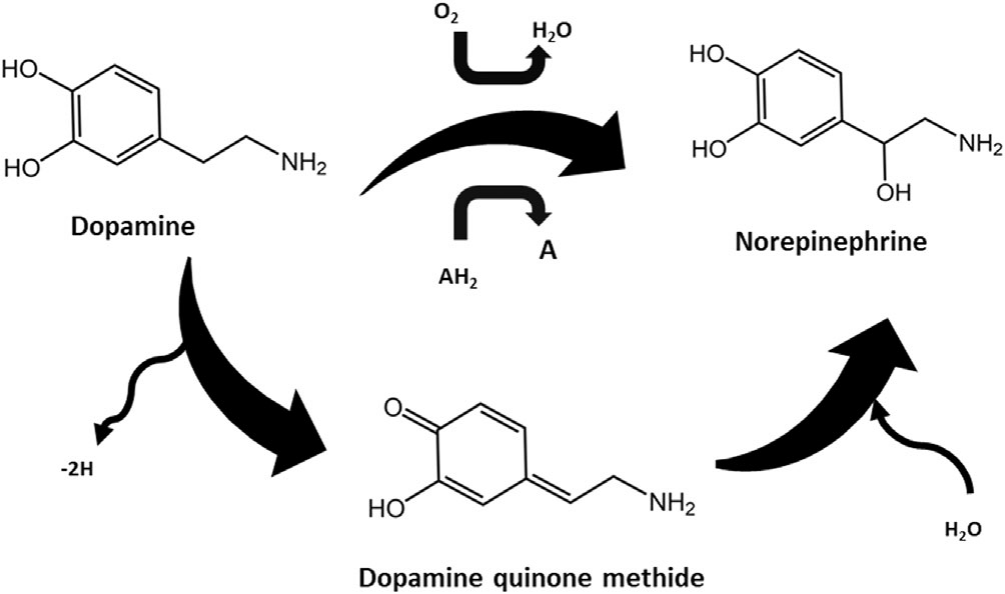
Schematic illustration of dopamine quinone methide

Crosslinking and reactivity of catechols strongly depend on the pH. In mild acidic environments (i.e., pH = 5.7–6.7), quinone methide, which is a highly reactive intermediate, becomes more stable and retard further reactions resulting in a slower rate of crosslinking.^98^ Under neutral to mild basic conditions (pH = 7.4–8), the rate of crosslinking reaction is high. In this range, quinone directly transforms to α,β‐dehydrodopamine, which may react with dopamine quinone, O_2_, or oxidization agent to yield its quinone form. At higher pH values, dicatechol species have been observed. Aryloxy radicals, which are generated by both catechol and quinine moieties, are responsible for the fast cross‐linking of catechol‐modified polymers.^99^ In quinone methide (Figure 12), there is one carbonyl oxygen substituted on the benzene ring. This makes it more polar and extremely reactive compared to quinone with two carbonyl oxygen. Para‐quinone methides are related to catecholamines.

## 7 CATECHOL‐BASED CHEMISTRY FOR MODIFICATION OF BIOMATERIALS

Catechol‐containing species may as side chains, chain caps, or it may be a co‐monomer. DOPA and other catechol‐containing chemicals induce wet adhesion and self‐healing properties to the polymers. Besides, they endow fast curing properties. Natural polymers and some synthetic polymers may be functionalized with catecholic derivatives as side chains or end‐caps. On the other hand, monomers or oligomers may also be functionalized with catecholic derivatives before polymerization to yield copolymers. Besides, catechol‐modified initiators may also be used to create catechol‐containing polymers.^39^

### 7.1 Premodification strategies

As discussed in the introduction section, the mussel's foot should provide a cavity of controlled condition before the secretion of MAPs. Adjusting the pH, oxygen concentration, and ionic strength are prerequisites for successful adhesion. In this regard, adjusting and controlling the conditions of the reaction medium is very important when designing and synthesizing catechol functionalized polymers and monomers or other catecholic compounds. Besides, several methods may be utilized to protect OH or amine in catechol groups during functionalization or polymerization. Protecting the α‐amino groups is critical in peptide chemistry. These protecting groups should be removed efficiently and fast while yielding easily removable byproducts.^100^ 9‐Fluorenyl methoxycarbonyl (Fmoc) and *tert*‐butyloxycarbonyl (Boc) group are the most frequently used protecting groups for α‐amino in peptide synthesis which are used in Fmoc/*tert*‐butyl (tBu) and Boc/benzyl (Bn) methods, respectively. In organic chemistry, 9‐fluorenylmethoxycarbonyl (Fmoc) is a base‐labile protecting group that is used for protecting α‐amino groups.^101^ In this regard, several protecting groups have been utilized to protect amino groups (e.g., Fmoc, Boc, carbobenzyloxy, acetyl, benzoyl, benzyl, and carbamate), the carboxylic acid groups (methyl esters, benzyl esters, *tert*‐butyl esters, and silyl esters), and phenolic hydroxyl groups (e.g., *tert*‐butyl ether, methoxymethyl, tetrahydropyranyl) after a modification with catechol. Acetyl,^102^ t‐butyldimethylsilyl chlorides (TBDMS‐Cl),^103^ cyclic ethyl orthoformate (Ceof),^101^ carboxybenzyl,^104^ acetonide,^105^, ^106^ methyl ether^107^ have been used as protecting groups for catechol during multistep organic synthesis methods.

Diethers or diesters can be used to protect catechol similar to strategies that are used to protect phenols from yielding cyclic esters, and cyclic acetals and ketals.^108^ Furthermore, several strategies (e.g., by using acids for Boc or bases for Fmoc) have been developed to remove these protecting groups after synthesis.^100^

### 7.2 Natural polymers

Natural polymers can be classified into polysaccharides, proteins, and polyesters. A beak mimic (which is based on DOPA) has resulted in manufacturing water processable chitosan composites.^109^

α‐Amino acid N‐carboxy anhydrides (NCAs) are reactive derivatives of amino acids that could be prepared through phosgene treatment.^104^ Polypeptides can be prepared through ring‐opening polymerization of NCAs. Copolymerization of NCA monomers of l‐lysine and l‐DOPA yields water‐soluble copolymers. After crosslinking with oxidants, these copolymers create adhesives that are resistant to moisture and show high adhesion to steel, glass, and synthetic polymers.^104^ Pluronic L‐31 will serve as an initiator of NCAs ring‐opening polymerization to yield thermosensitive hyperbranched poly(amino acid).^110^ However, there are a few research works on the functionalization of proteins with catechol, gelatin,^111^ silk fibroin,^112^ and collagen.^18^

**FIGURE 13:**
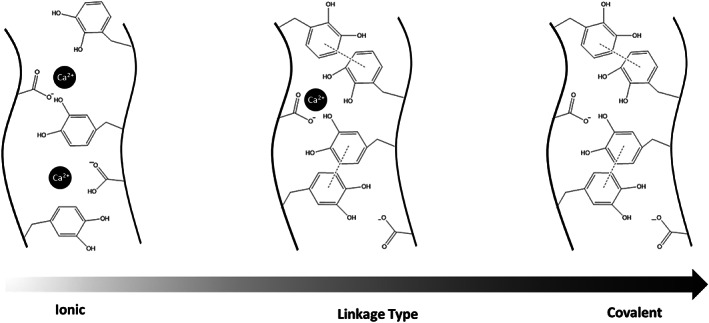
The crosslinking of catechol quinone in various conditions (ionic, linkage‐type, and covalent)

Polysaccharides such as alginate,^113^ chitosan,^114^ hyaluronic acid,^115^ dextran,^116^ chondroitin sulfate,^117^ and cellulose,^118^ have also been modified using catechol chemistry. Since these are water‐soluble polymers, the catechol modification is usually carried out using EDC/NHS chemistry to create side chains with catechol functionality using –NH_2_, OH, and COOH groups on the sidechains of polymers. Alginate is one of the most studied polysaccharides in biomedical applications. Unmodified alginate can immediately change into a gel in the presence of Ca^2+^ or other divalent cations because of ionic cross‐linking (electrostatic interactions) with the carboxylic groups.^119^ This phenomenon is fundamental for the encapsulation of drugs and cells. However, because of physical cross‐linking, the dissociation of ionic bonds between cations and carboxylic acid groups on the alginate is facile, resulting in ion dissolution.^119^ On the other hand, chemical crosslinking increases the physical stability of the gel, while chemical coupling agents can damage cells and proteins when encapsulating.^120^ Accordingly, a combined approach has been used in which cell encapsulation is triggered by an ionic coupling agent followed by a gradual substitution by covalent crosslinking.^121^ The gels' stability and mechanical properties (known as Stable Alginate Gel Prepared by Linkage Exchange [STAPLE]) increase gradually. STAPLE is based on the oxidation chemistry of catechol side groups that are gradually oxidized and create catecholquinone intermediates. As shown in Figure 13 when ionic bonds dissociate at physiological conditions (i.e., pH = 7.4 and 37°C), the crosslinking of catecholquinone occurs at a similar rate. Thus, gel maintains its integrity in contrast to common alginate hydrogels, which can “dissolve” in excess of water. The relative swelling for alginate–catechol gels was measured to be 660% compared to 350% for ordinary alginate gels.^121^

### 7.3 Synthetic polymers

Catechol may be used to manufacture copolymers, or it may be grafted to synthetic polymers. Besides, it may be used to synthesize end‐capped polymers.

DOPA was also used for various copolymers. Stable and biocompatible DOPA and lactide block and graft copolymers (i.e., PDOPA‐g‐PLA and PDOPA‐b‐PLA) can be generated using lactide ring‐opening polymerization in the presence of PDOPA, which also acts as an initiator.^122^ The LA:DOPA molar ratio, determine the molecular weight and composition of the obtained copolymers. It was observed that the thermal stability and degradation rate of graft copolymers are higher compared to block. Besides, increasing DOPA in copolymer results in faster degradation in which catechol functional groups bear oxidation and chemical crosslinking reactions. When polymerization of DOPA, it is possible for the amine groups to react with carboxyl groups such that the amine should be protected using Boc‐ or Fmoc‐strategies (i.e., Boc‐DOPA and Fmoc‐DOPA) in which Boc and Fmoc denote *tert‐*butyloxycarbonyl, 9‐fluorenylmethyloxycarbonyl. Besides, catecholic oxygen could be protected using labile acid groups such as *tert‐*butyldimethylsilyl (TBDMS).^101^ DOPA‐derivatives protected by Fmoc‐DOPA(TBDMS)_2_ were used to create DOPA‐modified peptides.^123^ Amine and hydroxyl groups of DOPA were protected by N‐(ethoxycarbonyl)phthalimide and acetyl chloride, respectively (Ac‐N‐Phth‐DOPA), to synthesize a hyperbranched PDOPA polyester.

The *T*_g_ of the polymer is 106°C, while its melting point is surprisingly higher than 200°C. the molecular weight was reported to be around 12,000 g/mol. High degradability and excellent biocompatibility were also observed. Cell adhesion of PDOPA was comparable to that of tissue culture polystyrene (TCP). In this study, pyrene and PLA have also been grafted to PDOPA. Reactive amine groups on the PDOPA enable its functionalization with other chemicals and polymers. Poly(ethylene) glycol (PEG‐g‐catechol) (with grafted catechol) can be prepared through polymerization of PEG in the presence of dopamine.^124^ The obtained copolymer can be used for the PEGylation of different surfaces and manufacturing antifouling coatings. Catechol acetonide glycidyl ether (CAGE) is a protected catechol derivative that has been utilized to endow catechol functionalities to hydrophilic polyethers.^125^ Both linear PEG and hyperbranched polyglycerol copolymers can be synthesized through the polymerization of CAGE with ethylene oxide and glycidol, respectively. PCAGE‐b‐PEG‐bPCAGE triblock copolymers create hydrogels with iron ions. Catechol‐containing chemicals can be used with polymerization initiators. Sadaba et al. synthesized end‐capped polylactide using dopamine as an initiator. They conducted lactide ring‐opening polymerization (ROP) in the presence of unregulated dopamine (i.e., without using protective groups). In some research studies, monomers or oligomers have been modified with catechol‐containing groups before polymerization. In another study, Lee and colleagues attempted to synthesize N‐methacrylated DOPA monomers and photopolymerized DOPA with poly(ethylene glycol) diacrylate (PEG‐DA) to create hydrogel under UV or visible light irradiation.^103^ Copolymerization of DOPA and PEG‐DA resulted in better improvement of mechanical properties of PEG‐DA for biomedical demands.

Glass et al.^126^ have introduced the synthesis of dopamine methacrylate (DMA), a monomer bearing the DOPA‐group in the side chain, which can be copolymerized with a variety of vinyl‐monomers easily. For example, Vatankhah‐Varnoosfaderani et al.^63^, ^127^ have synthesized copolymers of DMA and thermosensitive N‐isopropylacrylamide, which has proven to be a triple ion‐responsive polymer, able to form hydrogels as well as organogels with various metal and polymeric^128^, ^129^, ^130^, ^131^ ions and also through hydrogen bonding. Copolymers of DMA with methylmethacrylate were synthesized for their drug‐releasing and metal oxide absorbing properties, to be applied in hyperthermic cancer therapy.

### 7.4 Biomolecules

Peptides and proteins are created from amino acids and are essential components in cells. However, peptides are smaller compared to proteins, and they are structurally different. Oligopeptides (with 2–20 amino acids) and polypeptides are subgroups of peptides. Joining one or more polypeptides can result in forming proteins. DOPA‐modified short peptides have been synthesized using DOPA–DOPA (two DOPA attached through amide bond) and Fmoc–DOPA–DOPA species as raw materials. These peptides can self‐assemble to create fibers with high catechol groups. Besides, Fmoc–DOPA–DOPA can act as low‐molecular‐weight hydrogelators. Fmoc–DOPA–DOPA–Lysine tripeptide exhibited superior adhesion properties.^132^, ^133^, ^134^

Protein‐bound DOPA (PB‐DOPA) can be created through enzyme reactions in the mammalian cell.^135^ When oxidative damage in vivo, the creation of PB‐DOPA is enhanced significantly as a defense mechanism.^136^

### 7.5 Zwitterions

**FIGURE 14:**
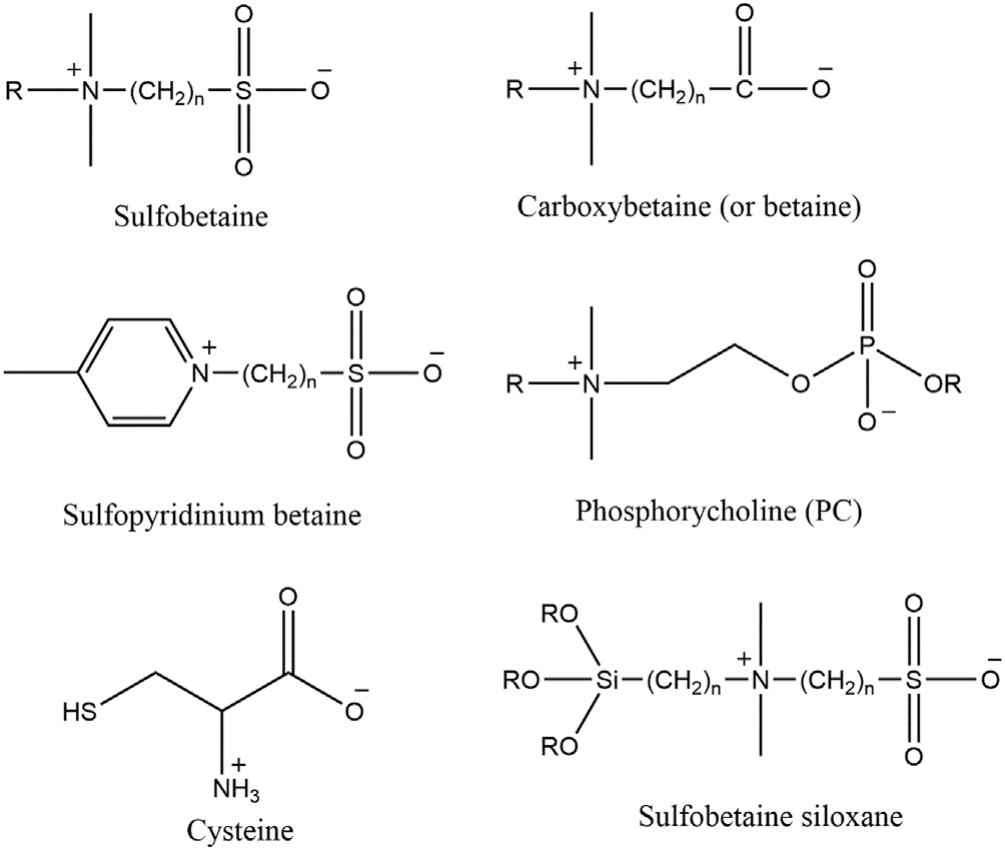
Schematic of Zwitterionic functional groups and one zwitterated siloxane^140^

Zwitterions are organic salts containing two or more functional groups, containing at least one positive and at least one negatively charged functional group.^137^ They have been used in biomedical applications.^138^ More interestingly, phospholipid as a major comprising component of cell membranes can be considered a zwitterionic derivative. The hydrophilic head of phospholipids is made of a covalently bonded pair of the cation (cholinium) and anion (phosphonate; Figure 14).^139^

**FIGURE 15:**
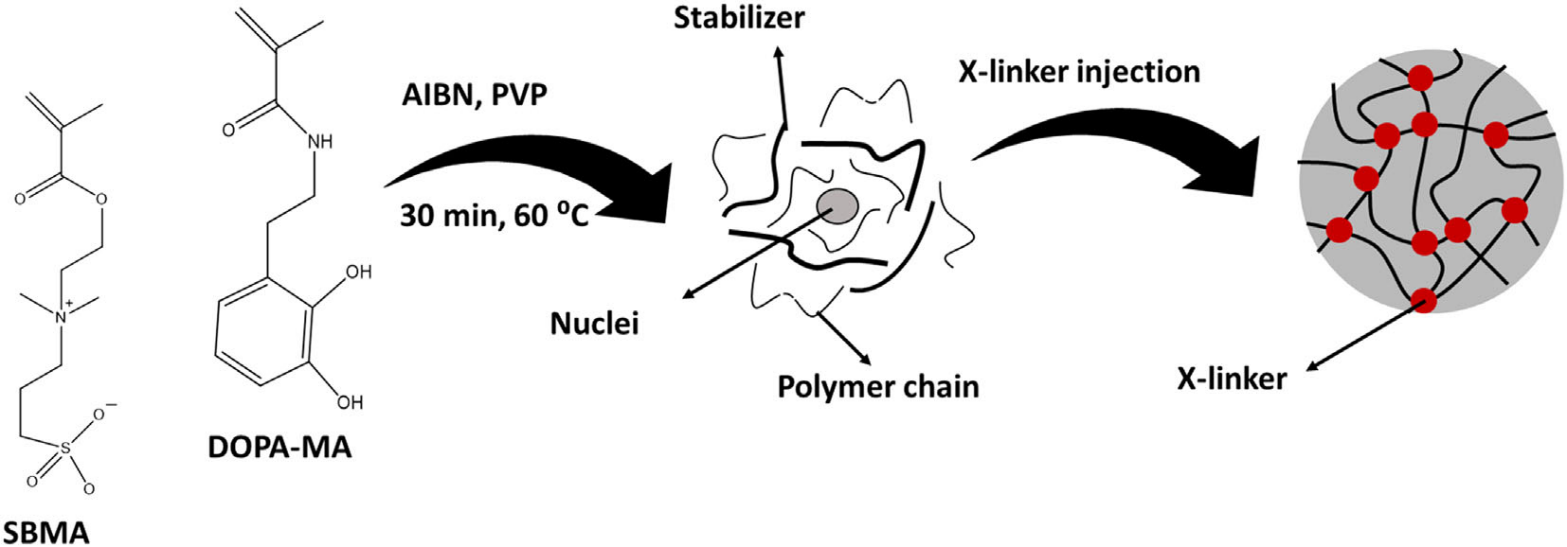
Microgelation of dopamine and zwitterion

Copolymers of zwitterionic and dopamine have been used for multifunctional coatings.^141^ They synthesized dopamine methacrylamide (DA‐MA) monomer and polymerized it in the presence of N‐(methacryloxypropyl)‐N,N‐dimethyl‐N‐(3‐sulfopropyl) ammonium betaine (SBMA), which is a zwitterion, to obtain microgels. They used AIBN as a polymerization initiator and PVP as a stabilizer. Besides, N,N′‐methylene bisacrylamide (BIS) was added as cross‐linker after the initial nucleation of the polymer, as shown in Figure 15, the resulting microgels have dual functionalities of dopamine and zwitterion. The former functionality, thanks to catechol chemistry, contributes both to the attachment of microgels (i.e., like an intraparticle cross‐linker) and adhering to diverse surfaces. The latter (i.e., zwitterion) enhances the water absorption capability on the surface, which results in antifouling and anti‐fogging characteristics.^141^

Wet adhesives based on catechol chemistry may be limited due to strictly controlled steps and complex and costly chemicals used when functionalization of polymers with catechol groups. However, catecholic zwitterionic surfactants containing catechol functional groups can be used as an alternative for MI wet adhesives with high adhesion performance.^142^

### 7.6 Modified nanoparticles/surfaces

Surface functionalization of substrate or particle may be done for different purposes. Enhancing interactions with matrix and dispersibility is one of the major reasons for the functionalization of nanoparticles. In this regard, functionalization nanoparticles using diverse functional groups such as alkyl, aryl, amine, carboxyl acids, phenols, and alcohols have been used. Catechol functional groups with diverse chemistry and various interactions can be utilized in the surface functionalization of different nanoparticles.

As discussed earlier, dopamine is a unique chemical that can undergo autoxidation polymerization and create a polymerized layer on every particle regardless of its surface chemistry.^5^ This allows surfaces such as noble metals, metal oxides, metals, semiconductors, ceramics, and synthetic polymers to be coated.^143^ This modification can enhance wettability, biocompatibility, enhanced cellular uptake, altered surface morphology, and cell affinity of nanoparticles.^144^ Besides, dopamine‐coated surfaces contain reactive moieties that can react with different materials. For example, biomolecules can be attached through nucleophilic interactions with amine and thiols, as discussed earlier.^31^, ^32^ Thus, the immobilization of various biological molecules such as DNA, drugs, peptides, proteins, and cells also on PDA‐coated inert surfaces is possible.^144^, ^145^, ^146^

Magnetic particles were used extensively in biomedical systems, including magnetic resonance imaging (MRI) contrast agents, hyperthermia, drug targeting, bioseparation, tumor detection, and ferrofluids.^147^ Ferrofluids, which are colloidal liquids of surfactant‐coated magnetic nanoparticles in an organic solvent or water, are usually used in biomedical applications. Iron oxide nanoparticles can be functionalized through catechol chemistry in which dopamine is a strong anchor for iron oxide. However, the reaction between Fe^3+^ and dopamine results in the degradation of iron oxide nanoparticles and limits its application as a ferrofluid.^148^ Ultrasmall superparamagnetic iron oxide nanoparticles (USPIOs) (diameter < 20 nm) can be used as a contrast agent for MRI. Catechol, phosphonates, and carboxylates are among the most commonly used anchoring groups for designing ligands for the surface of USPIOs.^149^, ^150^ Multidentate block copolymer (MDBC) or oligomers with catechol and other functionalities have been used to increase the performance of USPIOs.^151^, ^152^, ^153^ For example, multidentate ligands based on oligomers of poly(acrylic acid) containing several PEG derivatives and catechol groups were used to increase the biocompatibility and stability of Fe_3_O_4_ nanoparticles.^151^ Catechol groups firmly adhere to nanoparticles, while PEG functionalities improve nanoparticles' hydrophilicity, resulting in increased stability in a wide pH range (i.e., pH = 4–11) and under excess salt. The MIR results showed significant T2 contrast enhancement of these nanoparticles, while no cytotoxicity was observed for them.

Xiao et al. found that linear‐PEG/MDBC‐coated USPIOs have superior properties (i.e., improved signal performance in T1‐weighted imaging, high longitudinal relaxivities, good blood half‐lives [2–5 h], fast and efficient excretion in the liver and spleen in a few days after injection) compared to brushed‐PEG/MDBC‐coated counterparts.^154^ Thus, linear PEG moieties seem to be more promising when designing MDBC‐coated USPIOs. Besides, they inhibit protein adsorption on nanoparticles as well, which will be discussed then.

On the other hand, manganese carbonate nanoparticles coated with polydopamine have been used in magnetic resonance image (MRI)‐guided photothermal therapy (PTT).^155^ MR contrast is greatly improved when applying PDA coating because much more water molecules can be entrapped around nanoparticles. Lanthanide nanoparticles containing polymer coatings have been used for both diagnostics and therapies of cancers, while there are concerns about their biocompatibility and effectiveness. Coating with PDA (thickness ∼1.5 to ∼18 nm) can enhance biocompatibility and photothermal conversion efficiency.^156^ NIR‐II optical image and x‐ray CT dual images improve when the coating is applied.

Considering that the fate of nanoparticles in biofluids and cells is very important when assessing their effectiveness and possible side effects, engineering and designing the nanoparticles is of utmost importance to control their fate from entering the body through their clearance from the body. Cell/tissue targeting and cell uptake/trafficking of the nanoparticles depends on their physicochemical properties of them.^157^ Protein corona is a layer of proteins adsorbed onto various surfaces such as particles which results in disadvantages such as masking surface properties of nanoparticles (i.e., the chemical or biological functionalities that were deliberately imparted to nanoparticles) and providing nanoparticles with a biological identity that is detectable by the immune system.^158^ PEG coating has been utilized to control protein corona and cellular uptake of nanoparticles.^159^, ^160^, ^161^ Interestingly, the type of proteins that adsorb on PDA‐coated gold nanoparticles depends on the dopamine concentration.^144^

The diffusion of nanoparticles into the cells, known as cellular uptake, is critical in diagnostic and therapeutic applications. Besides, their fate in the physiological environment and the intracellular condition is of prime importance to do their functions effectively.^157^, ^162^ For intracellular applications, they are usually designed to deliver specific chemicals to the nucleus or cytosol. However, the diffusion of nanoparticles into the cells in a controlled manner is challenging. Cellular uptake of PDA‐coated gold nanoparticles is highly dependent on the polymerization time of dopamine. In other words, limited polymerization time results in enhanced diffusion of nanoparticles into Neuro‐2a and HeLa cancer cells. PDA coating can significantly improve the cell entering capability of nanoparticles compared to polymeric coatings.^144^ However, our knowledge about the nature of protein corona around coated nanoparticles with polydopamine is minimal, while it will significantly affect the fate of nanoparticles. There is a close relation between types of adsorbed proteins (corona), in the serum‐containing medium, and the concentration of dopamine, while it did not observe any meaningful relationship with polymerization time.^159^, ^160^, ^161^ More interestingly, serum‐stabilized Au@PDA nanoparticles enter the cells more easily while maintaining their morphology compared to Au@PDA prepared in a serum‐free medium.^144^

Multifunctional coatings can be designed and manufactured based on polymers containing different side groups where catechol provides adhesion to different surfaces. For example, antimicrobial coatings can be engineered using catechol, quaternary ammonium, and methoxyethyl side chains, which endow them with adhesion, antibacterial activity, and adjusting the amphiphilic balance, respectively.^163^

Titanium (Ti) is a biocompatible and inert metal with high corrosion resistance properties. These properties, along with high mechanical strength, have made it a promising bone substitute material. However, it has limited osteoconduction and osteoinduction capacities.^164^ The surface of Ti can also be modified by PDA‐coated Fe_3_O_4_ nanoparticles to enhance osteogenesis.^165^ PDA‐coated Ti implants, with enhanced corrosion resistance, higher cell viability, and lower contact angle was used in dental Implants applications.^166^ Besides, the integration of hard medical implants (e.g., Ti) with soft tissue and the wound healing process can be improved through a layer of gelatin hydrogel on a PDA‐coated implant.^145^

Silicon, due to its biocompatibility, low surface energy, smooth surface, and chemical, thermal, and biological stability, has been used in biomedical applications such as bio‐implants, coating for cardiac pacemakers, shunts, and microfluidic devices.^167^ However, there are still concerns, especially adverse immunological reactions, about the long‐term utilization of silicon‐based implants in the body.^168^, ^169^ Coating the implants with various biomolecules can reduce these concerns. Biocompatible PDA coating can adhere to the PDMS surface, increasing cell attachment, proliferation, and differentiation. Besides, the hemocompatibility of silicone‐based implants can be enhanced using PDA and hyaluronic acid (HA) coatings.^170^ Moreover, PDMS‐based organ‐on‐a‐chip devices can be more effectively designed using PDA coating, which can improve cell metabolism.^171^

Besides, polymeric scaffolds have been coated with PDA to improve its cellular interactions and immobilization of biomolecules.^172^, ^173^ This will be covered in the section on biomedical applications. PDA has been used as a general approach for bio‐surface modification.

## 8 BEYOND CATECHOL CHEMISTRY

Electrophilic substitution reaction with chlorine in para position results in stronger adhesion. This is observed in cement proteins secreted by sandcastle worms in which there is a remarkable amount of chlorinated derivative of DOPA.^174^ Such electron‐withdrawing groups, reduce the dissociation constants (p*K*_a_) of hydroxyl groups of the phenolic ring (which promotes catechol–metal complex at lower pH and higher stoichiometry) and decrease their redox potential which makes catechol oxidation harder.^175^ Similarly, substitution with the nitro group also enhances the adhesion and reactivity of catechol while improving the thermal and oxidation stability of catechol.^175^, ^176^, ^177^, ^178^, ^179^ Nitrodopamine can quickly cure and attach to biological surfaces in acidic pH (compared to dopamine), making it a promising candidate for manufacturing bioadhesives for acidic tissues.^176^ Increased degradation rate, degradation by light, and reducing p*K*_a_ are other properties induced by nitro substitution.^178^, ^179^, ^180^

**FIGURE 16:**
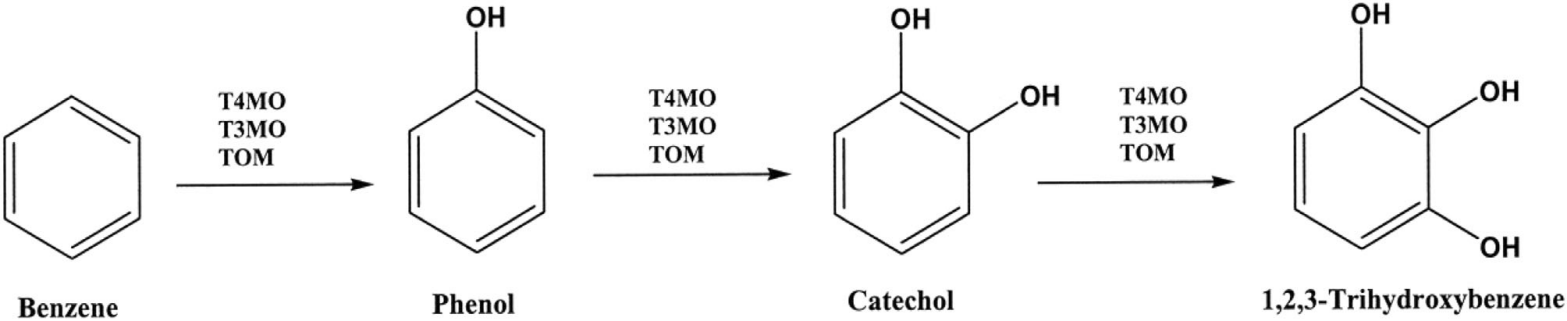
Synthesis route for trihydroxybenzenes from the benzene ring

Adding one extra OH group to the benzene ring makes trihydroxybenzenes, which has a remarkably enhanced tendency toward complexation with metal ions and boronic acid as well.^181^, ^182^ As discussed earlier, this characteristic is utilized by some bacteria siderophores for iron acquisition in scarce environments. Pyrogallol or gallol is a trihydroxy benzene with three vicinal hydroxyl groups on the benzene ring (Figure 16). In other words, this organic compound has one more OH relative to catechol. Like catechol, pyrogallol is an allelochemical, that is, produced by living organisms. Despite all structural similarities between pyrogallol and catechol compounds, they exhibit different characteristics. For example, the antioxidant activities of gallol‐conjugated compounds are higher than that of widely used catechol‐functionalized materials. Moreover, it is suggested that catechol‐containing platforms show both antibacterial and antifungal properties while pyrogallol only may be effective against only bacterial diseases. Tannic acid contains pyrogallol. Both catechol and gallol show antimicrobial properties and antioxidant radical scavenging activity.^183^, ^184^, ^185^

Tunicates (subphylum Tunicata) are a kind of marine invertebrate species, including sea squirts and salps but not jellyfish. Their tough and flexible outer covering (i.e., tunic) is secreted from the body wall. Its major components are cellulose nanofiber (CNF) and proteins containing TOPA (trihydroxyphenylalanine) which include gallol functionality.^186^, ^187^ Gallol groups, like catechol, can chelate metal ions and perform chemical cross‐linking reactions such that they can contribute to wound healing of tunicate. This makes tunicate‐inspired (TI) chemistry for designing bioadhesives. Using TI coupled with MI chemistry (i.e., catechol and gallol functionalities), Zhan et al.^187^ manufactured an underwater adhesive. They synthesize a family of poly(vinylgallol‐co‐*n*‐butyl acrylate) [p(VGal‐co‐BA)] copolymers. Interestingly, the designed bioadhesive copolymer is seven times stronger than catechol‐functionalized copolymers. Besides, its performance in seawater is superior compared to isocyanate‐based adhesives.

## 9 BIOMEDICAL APPLICATIONS

### 9.1 Catechol–tissue interactions

**FIGURE 17:**
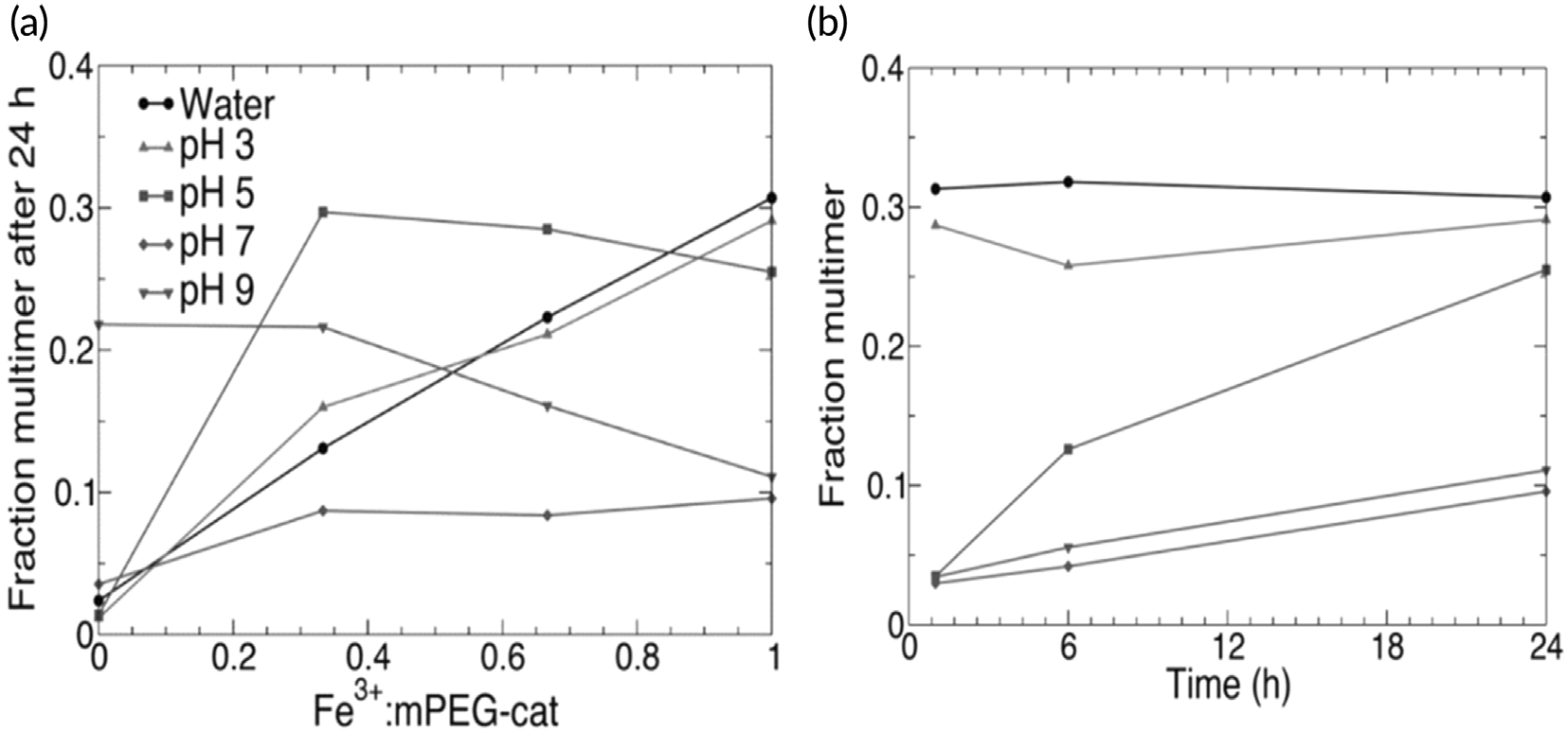
Correlation between pH in the polymerization process of catechol‐containing polymers and the degree of polymerization (a). Multimer fraction versus time at a 1:1 Fe^3+^:catechol ratio (b)^188^

As discussed before, various mechanisms of cross‐linking such as metal complexation and oxidative, incorporated with different influential factors, including pH, and the concentration of oxidants are controlling the kinetics, catechol reactivity, and crosslinking ability of systems. The catechol reactivity and crosslinking ability depend on the pH of the environment. Barrett et al.^188^ investigated the relationship between the reaction pH and behavior of materials created by reacting ferric ions (Fe^3+^) with synthetic catechol polymers. In this work, gel‐permeation chromatography (GPC) validated the polymerization phase of catechol‐containing polymer (PEG) (mPEG‐cat) has been slowed down, as the pH of the reaction is increased from pH = 3 to pH = 5. The mPEG‐cat polymerization degree was significantly higher at pH 5 after 1 day of reaction. By raising the pH value to 7, the outcomes of the polymerization process, as well as the multimerization, were qualitatively comparable to those at pH = 5, albeit a slower‐moving rate of crosslinking was reported. At both pH 7 and pH 5, the amount of multimer fraction after 24 h of reaction was independent of the Fe^3+^:catechol ratio, when this ratio was in the range of 1:3–3:3 (Figure 17a). After 1 day, all of the mPEG‐cat solutions with Fe^3+^ contained 25%–30% and 10% multimer for the reactions that happened at pH 5 and pH 7, respectively (Figure 17b). Also, four‐armed PEG bioadhesive with dopamine end‐caps strongly depends on the pH. Briefly, in the mildly acidic region, due to the increase in the extent of stabilized transient oxidation intermediates, a slower curing rate of four‐armed PEG was observed, while under basic conditions (pH = 8), it was cured quickly.^99^ Thus, when catechol‐containing hydrogels or bioadhesives are used in vivo, they may undergo different reactions depending on the physiological pH levels. From the body tissues' point of view, the pH ranges for skin, subcutaneous tissue, and dysoxic tissue (due to blood loss) are 4–6,^189^ 6.7–7.1,^190^ and 7,^191^ respectively. For oxygenated blood and internal tissues, pH is 7.4, while tumor tissues and skin are acidic.^99^

### 9.2 Scaffolds

Polymeric scaffolds were commonly used in tissue engineering and regenerative medicine. Scaffolds, as a temporary matrix, primarily provide the cells with structural support and an adequate microenvironment to accomplish any adhesion, development, proliferation, and differentiation measures, just like the native ECM.^192^ Different polymer materials with various structures may be used to fabricate scaffolds. Selecting proper polymers are based on their unique properties such as the chemistry of polymers, average molecular weight, the length of chain prolongation of polymer, hydrophilicity, and hydrophobicity, solubility in various solvents, the adsorption capacity of H_2_O, biocompatibility, erosion mechanism, and biodegradability.^192^, ^193^ Also, biodegradation feature, biocompatibility, physical structure (i.e., 2D or 3D skeleton, tailorable surface: volume ratio and tunable porosity), chemical structure (e.g., the versatility of chemistry, constructing materials, and embedded biomolecules), mechanical properties (e.g., stiffness, modulus), degradation (e.g., degradation rate and products), or the biological properties of the scaffolds directly affect cell behavior and viability.

3D scaffolds may be designed based on fibers, hydrogels, or sintered particles. Electrospinning processes were used to fabricate PCL/gelatin nanofibers, followed by crosslinking and surface modification using PDA.^194^ PDA coating results in surface roughness on nanofibers decreasing and hydrophilicity increasing (i.e., water contact angle declines). The obtained nanofibrous membranes were cultured with adipose‐derived stem cells (ADSC) from mice where cell adhesion, spreading, and viability was improved. After cell culture and incubation, three membranes were stacked through layer‐by‐layer (LBL) assembly to create 3D scaffolds. It was also observed that cell growth and ADSC differentiation to osteoblasts are improved for PDA‐modified 3D scaffolds. Nowadays, the application of PDA as a coating agent to level up the characteristics of prefabricated 3D scaffolds for better cell adhesion, growth, and proliferation, is a hot research topic.^195^

Razavi et al.^196^ manufactured collagen‐based hydrogels at sub‐zero temperatures (i.e., cryogel) and coated the obtained scaffolds with PDA. It was observed that water absorption, swelling rate, and degree of dissolution of the scaffolds decrease while their mechanical properties (i.e., stiffness and compression strength) increase upon PDA coating. The survival and multiplication of Adipose‐derived mesenchymal stem cells (ADMSCs) for coated scaffolds are higher. These findings suggest, for example, that PDA‐modified cryogels may be used as a matrix material for storing and transporting ADMSCs in cell therapy applications.^196^

3D printing technology is frequently used in the biomedical engineering field, especially in engineered bone tissue, since many printable biomaterials and biopolymers are designed and manufactured in this area of research. 3D printers have attracted great attention in tissue engineering applications, especially manufacturing polymeric scaffolds, because they can overcome some limitations of traditional methods.^197^ Besides, bioactivity and cellular interactions of the designed scaffolds may be increased via the incorporation of catechol‐containing moieties.^198^ Based on this idea, PDA‐coated 3D printed PLA scaffolds with enhanced bioactivity were fabricated using a relatively simple one‐step procedure.^199^

Furthermore, PDA coating facilitates the immobilization of type I collagen (COL I) onto the surface. Surface morphology was significantly changed such that surface roughness decreased upon coating. The ECM deposition on scaffolds, osteoinductivity of scaffolds, cell adhesion, and metabolism (bone marrow stem cells of porcine) in an early stage of culturing improves when the scaffold is coated by a COL I‐PDA layer. Not only the catechol functionalities can significantly promote the adhesion of the cells, but also they can profoundly enhance the spreading of cardiomyocyte progenitor on supramolecular surfaces, which could be potentially applied in cases related to cardiac tissue damages.^200^ Mai et al.^201^ designed and prepared a bioceramic scaffold using a 3D printing technique for bone cancer treatment and tissue regeneration. By taking into account the benefits of using biocompatible PDA with outstanding biodegradability and its elevated photothermal capability, they prepared uniform PDA nanolayers via its self‐assembling process onto an as‐prepared 3D bioceramic (Nagel) scaffold. This scaffold provided an exquisite environment for osteoblastic stem cells of rabbit bone to attach, differentiate, and proliferate. According to this report, the regeneration of bone tissue was highly accelerated and desired, whereas photothermal therapy was in progress.^201^

### 9.3 Biointerfaces

**FIGURE 18:**
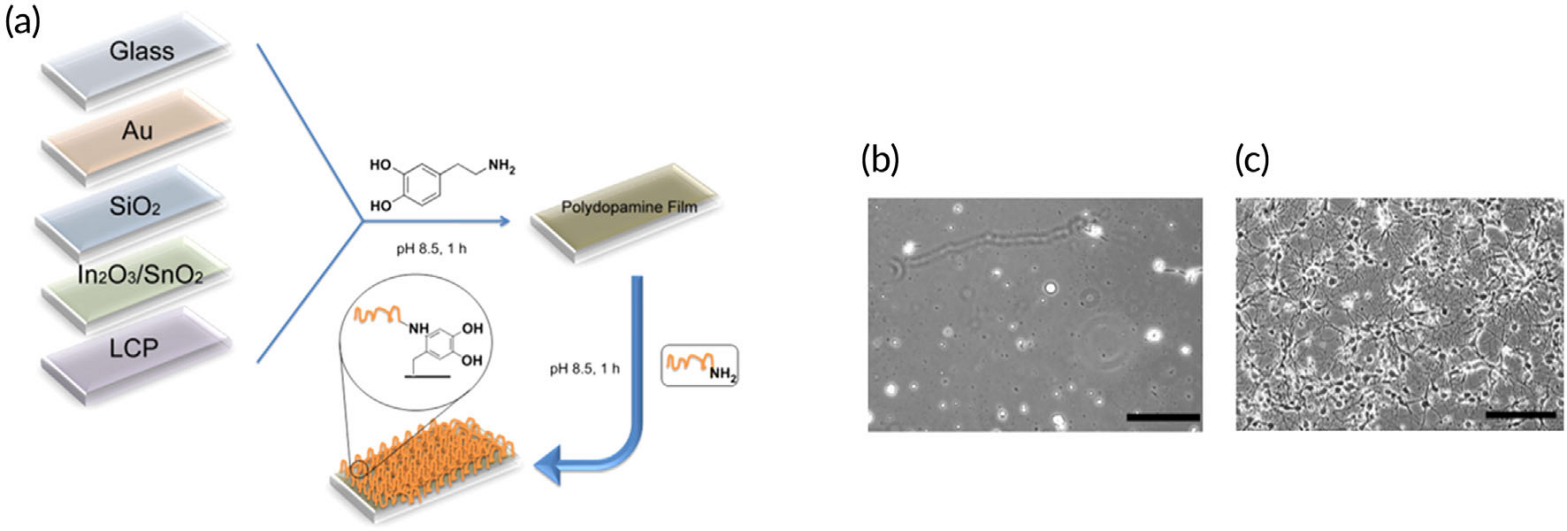
Schematic illustration of polydopamine coating of different substrates and subsequent conjugating of amine molecules (a). Hippocampal neurons are cultured on PDA‐modified substrate (b) and polylysine immobilization PDA (c). Scale bar^207^

In several applications, such as neural prostheses, neurons on a chip, and tissue engineering, the interfacial contact between neuron cells and other surfaces is inevitable, which results in inflammatory responses.^202^, ^203^, ^204^ Consequently, surface modification strategies (e.g., immobilization of biomolecules on the surface) have been utilized to decrease such responses.^201^ However, several metals (e.g., gold, stainless steel, platinum, titanium, tungsten) and insulating materials (e.g., parylene, polyimides, SU‐8, iridium oxide, and titanium nitride) have been utilized for the development of neural probes and arrays.^205^, ^206^ However, modification strategies are still needed, especially for prolonged usage. PDA as ubiquitous coatings could be applied to modify neural interfaces. Using this strategy, Kang et al.^207^ coated the surface of a variety of neural interfaces by PDA, followed by polylysine immobilization through chemical crosslinking. In neuron culture experiments the neuron does not survive on PDA, while polylysine‐linked PDA shows good viability and growth for neurons that form neuronal networks. Planar microelectrode arrays (MEA) were coated by polylysine‐linked PDA coating. These coated MEAs can adequately record the neural signals. Thus, PDA coating does not interfere with the electrode function indicating that PDA is a promising material for neural electrode surface modification. After that, these researchers adapted the PDA deposition system and used an electrochemical deposition system (with +0.5 V applied voltage) to render PDA films.^208^ The electrochemical method was performed under mildly acidic conditions (i.e., pH = 6), while the solution polymerization of dopamine routinely is performed under basic conditions (pH = 8.2). Inherently conductive polymers (ICPs) were used to modify electrode surfaces for various applications, such as storing energy and biomedical applications.^209^ However, the poor adhesion of ICP films on the electrode is a challenging issue that limits their application. A possible solution is to utilize catechol chemistry to overcome this problem. The electrochemical deposition technique has been utilized to fabricate a composite coating containing PDA and polypyrrole.^210^ The adhesion strength of PPy was greatly enhanced while electrochemical impedance was significantly reduced, indicating the potential of this strategy for manufacturing high‐quality electrodes for various applications, including neural electrodes. In another study, to robust the differentiation of neuron‐like PC12 cells, norepinephrine (NE), a common moiety in both mussel adhesive proteins and neurotransmitters, was introduced as a specific and multi‐functional bio‐interface integrating agent to the surfaces of poly(l‐lactic acid‐co‐ε‐caprolactone) (PLCL) fiber‐based scaffold fabricated by electrospinning technique.^194^ Poly(norepinephrine) coated PLCL (pNE/PLCL) indicates a high capacity as a substrate for differentiation of PC12 cells (similar to neurons) and improves the practical use of growth factor, pharmaceutical agents, and other bioactive molecules with a lower dosage, leading to a decrease in the side effects of both drugs and drugs (Figure 18).^211^

### 9.4 Organic/inorganic composites

Combination with organic/inorganic compounds to create robust biomedical applicable materials, mussel‐inspired chemicals represent a pretty new, critical challenge. According to the literature,^212^, ^213^ a vast range of bioactive minerals hybrid composites (e.g., calcium‐based ones) has been fabricated using biomaterials secreted from mussels for regenerative medicine uses, especially for therapy application in teeth and bone tissue defects. Many researchers in the bone defections field, by exploiting nontoxic, adhesive biomaterials that can emulate mussel‐inspired proteins (especially catechol‐containing PDA), tried to coat the concentrated calcium cations onto various kinds of platforms. Generally, the flat and clean substrates such as ceramics (SiO_2_ glass), polymers (polystyrene, poly[methyl methacrylate], polydimethylsiloxane), semiconducting material (Si_3_N_4_), and noble metal and metal oxides (Ti, Si, Au, TiO_2_) have been used to enhance and facilitate the formation of the skeletal frame by formation of natural inorganic crystals of hydroxyapatite. Kim and Park^214^ introduced a new methodology to stabilize the synthesis of vaterite spheres with the aid of the oxidative polymerization of dopamine onto flat clean substrates while the CaCO_3_ gets biomineralized. Their findings indicated that taking a different route containing PDA, as an excellent biocompatible, bioresorbable polymeric platform for carbonated hydroxyapatite formation, could be immensely effective and impressive for the engineered bone tissue abnormalities and bone regenerative applications. Also, reports justify that the hydroxyapatite crystals have been applied to treat bone infections and for cancer therapy, as an effective drug delivery system.

**FIGURE 19:**
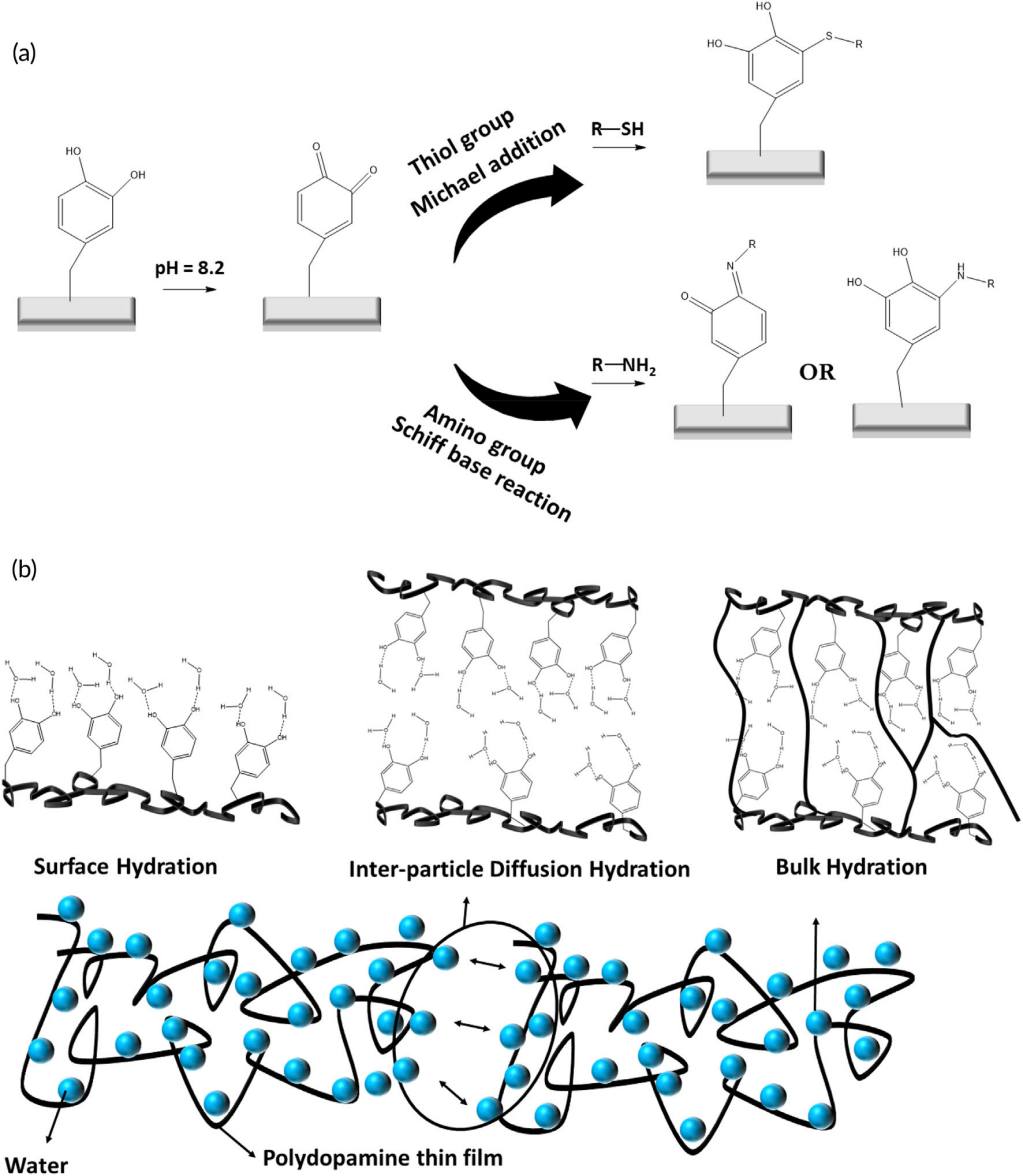
A different mechanism of (a) catechol group reaction, (b) Polydopamine hydration^216^

According to the previous reports, catechol conjunction and polydopamine coating enhance the feasibility and biocompatibility of organic and inorganic materials applied in the various fields related to medicine such as cancer therapy, wound healing, regenerative medicine, and drug delivery. Catechol conjunction ensures the needed hydrophilicity of materials for better recapitulation of real tissue conditions via hydrogen bond. The o‐quinone groups formed from oxidative catechol groups in polydopamine at pH > 7 and bind with the surfaces which contain amine or thiol groups through Schiff‐base reaction or Michael‐type addition.^215^ Polydopamine exhibits negative and positive charges at pH ranges above and below 4, respectively. Polydopamine can increase the hydrophilicity of materials via its various hydration mechanism (Figure 19).^216^ Furthermore, in the alkaline region, quinone groups facilitate the immobilization of different biomaterials like peptides, proteins, and growth factors, which contain amino or thiol groups. All in all, the outstanding features of catechol and polydopamine, such as excellent interaction with cells and high mechanical properties, lead to an increase in the application of these materials in biomedical engineering.

**FIGURE 20:**
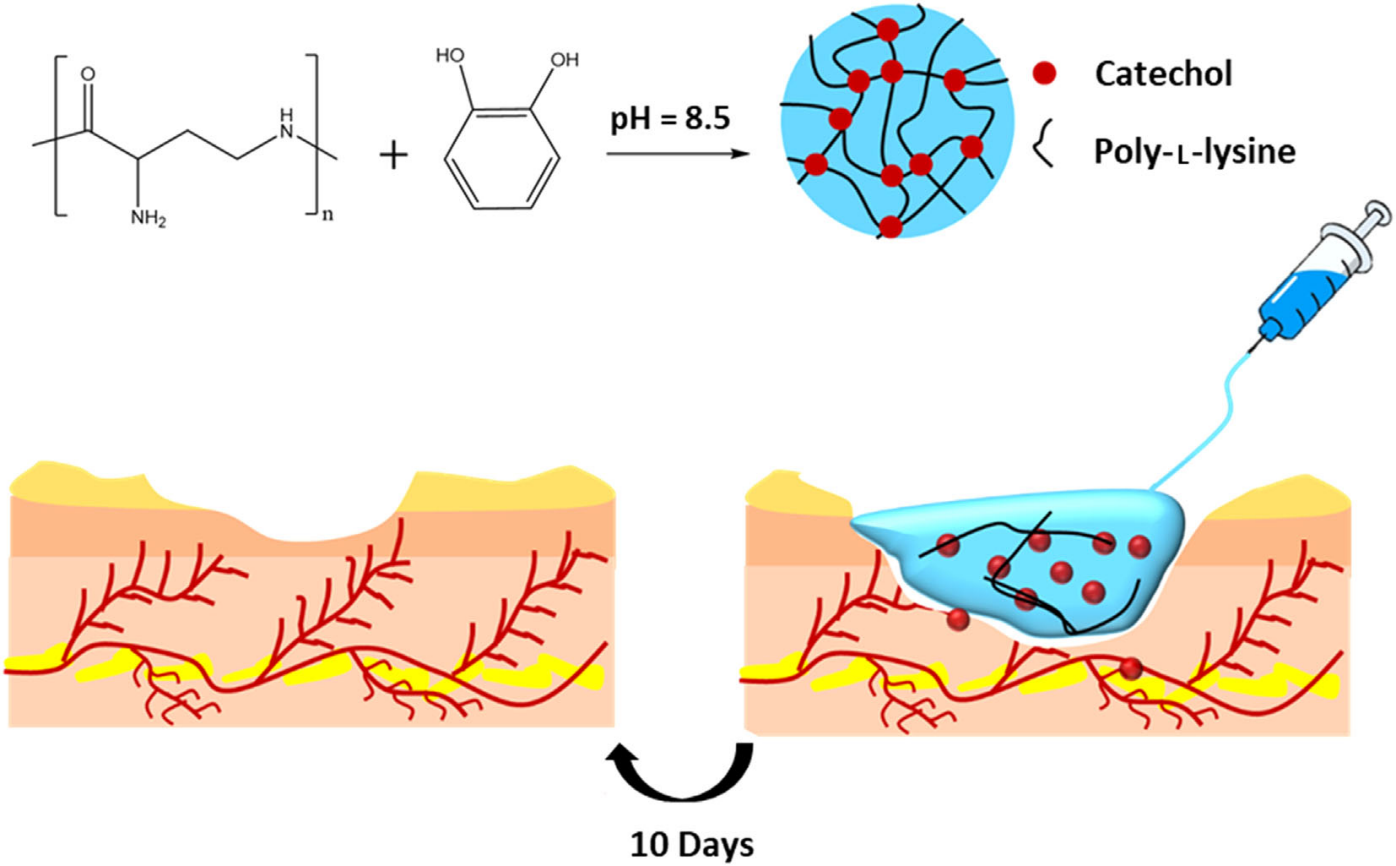
Representation of preparation of the composition of ε‐poly‐l‐lysine and catechol and its application for tissue engineering

A combination of biodegradable and adhesive poly(l‐lysine)/polydopamine with hydroxyapatite leads to better cell migration, proliferation, and protection as well as sustained release of the bioactive BMP2 as an osteoinductive growth factor.^217^ The interaction between hydroxyl molecules of dopamine which are immobilized on bacterial cellulose surface and rGO/Ag particles leads to the fabrication of a strong antimicrobial and wound‐healing dress. The in vitro study exhibited that the existence of rGO and Ag particles enhanced the regeneration process.^218^ Recently, researchers found that the composition of ε‐poly‐l‐lysine and catechol at mild condition by oxidative crosslinking at pH = 8.5, show desired antibacterial properties versus *Escherichia coli* and *Staphylococcus aureus*, minimizing the pro‐inflammatory cytokines, and enhanced tissue regeneration (Figure 20).^219^ Similarly, combining methacrylamide dopamine, 2‐(dimethylamino)ethyl methacrylate, and quaternized chitosan made the hydrogel contact‐active antimicrobial against both Gram‐negative and Gram‐positive bacterium. The presence of interior cross‐linking of dopamine in the complex increases its mechanical properties, toughness, and cell/tissue affinity.

### 9.5 Hydrogels

High‐water polymeric hydrogels and tissue‐imitating mechanical properties were used as cell attachment, growth, and delivery scaffolds. They also have numerous biomedical implementations, including drug/gene delivery, wound management, soft contact lenses, tissue engineering, and hygiene products.^220^ Until now, different kinds of natural and synthetic hydrophilic polymers were used to make hydrogels. Stimulus‐responsive hydrogels belong to a wide range of smart hydrogels that can be adjusted using different stimuli such as temperature, pH, and light.^221^

Injectable hydrogels that could be efficiently delivered to the target site using a syringe are very interesting for minimally invasive therapies.^222^ The injectable hydrogels may also carry cells, genes, or theranostic agents. When injected into the body, they experience phase transformation (i.e., sol–gel transition) as a result of in vivo stimuli such as temperature and pH. Self‐healing ability may also help the hydrogel to maintain its integrity in vivo where mechanical stresses are present. The toughness, mechanical strength, wet adhesion target tissue, and cytocompatibility of the hydrogels are also very important factors when applied in vivo. Besides, the absence of chemical crosslinking agents, which may be suspicious of cytotoxicity, and also the absence of metallic ions may enhance the hydrogel applicability.

Furthermore, wet adhesion properties of hydrogels are essential when used in the human body where water‐based biological fluids (e.g., extracellular fluid) make up a significant portion of total body weight. Wet adhesion allows the hydrogel to be fixed at the target area and prevents its movement upon exposure to frequent mechanical stimuli in vivo. MI chemistry can be used not only in designing wet adhesive hydrogels but also can endow hydrogels with self‐healing properties. Besides, MI chemistry allows us to design and manufacture stimuli‐responsive hydrogels. The thermosensitive nature of pluronic has had with high biocompatibility and water absorption of hyaluronic acid (HA) in manufacturing thermosensitive and injectable tissue adhesive hydrogels based on catechol‐thiol chemistry.^223^ Thiol‐capped pluronic F127 and dopamine‐modified HA can create a highly crosslinked hydrogel based on Michael's addition of catechol and thiol moieties. The obtained hydrogels show long‐term in vivo stability (mice model), superior tissue adhesion and mucoadhesion, and temperature‐induced gelation, which is appealing in tissue engineering and drug delivery application.

In addition, bio‐fouling in biological fluids is detrimental to hydrogels. Li et al. developed injectable, self‐healing, and thermosensitive hydrogels with anti‐fouling properties.^224^ Using a RAFT polymerization technique, they fabricate an ABA tri‐block architecture in which catechol conjugated PNIPAM is the A block (thermosensitive block) while poly(ethylene oxide) (PEO) creates the B block (hydrophilic block). The self‐assembly of the synthesized ABA triblock result in a thermosensitive hydrogel. PEO segments, endow the copolymer with antifouling characteristics which inhibit the adhesion of nonspecific cells. More fascinating, when temperature increase beyond LCST, the hydrophobic domains within PNIPAM provide a local hydrophobic microenvironment to preserve catechol from oxidation. This is similar to MFP‐3s in blue mussels in which high content of hydrophobic amino acid residues creates a local microenvironment and protects DOPA moieties from oxidation, as discussed in Section 1.

Tannic acid discussed earlier is rich in catechol and gallol functional groups. Abundant hydroxyl groups enable it to make a hydrogen bond with hydrogen‐bond donor polymers such as polyvinylpyrrolidone (PVP). Physically crosslinked reversible hydrogels can therefore be designed, showing pH‐dependent switching. In other words, as the oxidation of catechol to quinone depends on pH, hydrogen bonds forming and dissociating are also pH‐dependent, resulting in a reversible and dynamic hydrogel. According to this idea, pH‐responsive hydrogels were manufactured using tannic acid PVP, which exhibits shear‐thinning, self‐healing, and quick self‐recovery properties, which are prerequisites for injectable hydrogels.^225^ Furthermore, catechol and gallol functionalities enable metal complexation such that these hydrogels can be further cross‐linked using Fe^3+^ ions, resulting in a dual‐responsive reversible hydrogel. Avoiding chemical crosslinkers indicates these hydrogels' high biocompatibility for applications in various biomedical fields.

Chitosan is a mucoadhesive polymer that can be used for drug delivery, including Buccal mucoadhesive systems.^226^ In addition, MI chemistry can enhance the mucoadhesiveness of chitosan and its derivatives. In this regard, the mucoadhesive hydrogel was fabricated based on chitosan modified with hydrocaffeic acid as catechol‐containing chemicals.^114^ Besides, genipin was used as the crosslinker, which is a naturally occurring aglycone with low cytotoxicity.^227^ Contrary to neat CS hydrogels, which are released from the porcine mucosal membrane only after 1.5 h, the catechol‐functionalized CS exhibited long‐lasting adhesion. CS‐cat hydrogels were successfully used in a sustained buccal drug delivery system on the rabbit model.^114^

### 9.6 Wound management

Skin is the body's largest organ, comprising several layers of ectodermal tissue. The skin provides a controlled condition for the underneath organs, tissues, and cells while protecting them from harmful germs and toxic chemicals. However, it may be damaged, resulting in different problems such as bleeding and infection. Thus, caring for the incisions created through trauma, burn, and surgery is critical in healthcare. The wound may also be created by surgery. Over 312 million surgeries are estimated in 2012.^228^

Wound closure is usually done through suture. However, deforming the tissue, blocking the blood flow, remaining residual forces even after removing the suture, air/biofluids leakage, microbial infection, foreign body reactions that impede healing, being painful, damage to surrounding healthy tissue, poor cosmetic outcome, and being impossible in conditions are some of the disadvantages related to conventional sutures and staples.^229^ In this regard, different groups of tissue adhesives have been developed for surgery and tissue engineering applications.^230^ This adhesive is more easily applied and can be absorbed gradually. Besides, they are more economical, remove residual forces, act as a sealant by preventing fluid leakage, and possess better cosmetic outcomes.

Tissue glues are classified as hemostatic agents, sealants, and adhesives according to their function, which is expected to hit USD 7.4 billion in the year 2022. Hemostatic agents such as collagen‐based and gelatin‐based topical hemostats, only act in the presence of blood. The sealants, such as fibrin and protein‐based sealants, create a barrier layer to prevent fluids leakage, such as gastrointestinal anastomosis,^231^ and adhesives are used for bonding biological surfaces.^232^

Hemorrhaging (bleeding) is one of the significant problems associated with blood leakage from blood vessels. Massive hemorrhage can cause serious health problems and even death. Thus, preventing blood loss is very important in these cases. A process that causes the bleeding to discontinue is known as Hemostasis, which is considered the first step in the wound healing process. The various hemostatic agent has been developed based on a different mechanism of action which are commercially available.^233^ Blocking the blood flow through strong adhesion to the tissue surface is the main mechanism of action of some hemostatic agents. However, strong adhesion under the wet condition of bleeding can be quite challenging. The MI chemistry has been considered as a potential for this problem. Catechol‐modified chitosan has been used in manufacturing hemostatic swabs to stop bleeding.^234^ PDA coated silica nanoparticle was also developed for bleeding control.^235^ These antibacterial hemostatic agents show good degradation as well. Hemostatic agents based on catechol‐modified chitosan and thiolated poloxamer hydrogels show strong adhesion to soft tissues and mucous as well.^236^

A multilayer polysaccharide‐based membrane modified with catechol has been used to modify skin wound healing.^237^

Alginate‐based hydrogels have also been used as MI tissue adhesive for wound dressing. For example, hybrid hydrogels containing dopamine‐grafted oxidized sodium alginate (OSA‐DA) and polyacrylamide have been used in wound dressing applications.^238^ The synthesized hydrogel is very tough and shows self‐healing properties. The hydrogel toughening originates not only from chemical interactions between OSA and PAAM, but also the physical interactions between catechol moieties in side chains of OSA‐DA. In a similar work, OSA‐DA was utilized as a novel crosslinker for PAAM/collagen hydrogel systems, which are used in wound healing applications.^239^ Catechol functionalized chitosan with superior mucoadhesive and enhanced antibacterial activity (compared to pure chitosan) can be synthesized using hydrocaffeic acid.^240^ Antioxidant properties were also observed for this dressing which makes it more interesting for wound care applications.

PNIAPAM‐polydopamine nanoparticles hydrogels were also applied to wound healing.^241^ These thermosensitive hydrogels show improved cell affinity, self‐healing properties, and tissue adhesive properties, and they can immobilize epidermal growth factors as well. When imposing NIR radiation, the composite hydrogels show volume contractions, which indicate a photothermal phase transition, while similar behavior was not observed for pristine PNIPAM hydrogels. This contraction was used in drug delivery too.

Healing chronic wounds like pressure sores, diabetic foot ulcers, chronic venous ulcers, and arterial insufficiency are very difficult.^242^ The healing of chronic wounds is inhibited because of constant agitation of the inflammatory system induced by protease enzyme, reactive oxygen species (ROS), and exudates. Diabetic foot ulcers are the most common chronic wounds which impose a high cost on the healthcare system of countries. Approximately two‐thirds of amputations are in diabetic patients.^243^ The high concentration of protease can degrade different species such as elastin, collagen, and growth factors while ROS oxidizes biomolecules followed by triggering the inflammatory system. It was reported that naturally derived phenolic compounds show antioxidant properties.^244^ This antioxidant behavior was also observed for different catecholamines. Thus, catechol‐containing chemicals may also show antioxidant behavior along with wet adhesion. Hydrocaffeic acid (HCA) grafted to random copolymers of N‐vinyl caprolactam (V) and 2‐hydroxyethyl methacrylate was used as a bioadhesive resorbable membrane for chronic wound healing. Catechol‐containing HCA side chains provide wet adhesion for the copolymer. The biocompatible membrane shows an anti‐inflammatory effect and ultraviolet screening properties as well.^198^

Conjugated N‐vinyl caprolactam (V) and 2‐hydroxyethyl methacrylate (H) with catechol to produce a biocompatible tissue adhesive for wound healing.^198^

The scar is a fibrous connective tissue that may replace the healthy tissues after the wound heals. In scar tissue, small collagen bundles are aligned parallel to each other while normal tissue is composed of collagens with a random basketweave structure.^245^ Jeon et al.^246^ fabricated a scar‐preventing glue based on collagen type I and mussel adhesive protein (MAPs). The glue improves wound healing at an early stage (i.e., accelerated regeneration) and successfully prevents scar formation by adjusting the growth of collagen fibrils and fibrogenic factors expression. PDA‐modified polymers have also been used to develop tissue adhesives for wound closure. A variety of catechol‐containing poly(amidoamine) (CPAA) and catechol‐containing poly(amino ester) (CPAE) with different degradation rates were produced by adding Michael reactions.^247^ To produce CPAE, PEG diacrylates with a different number of EG units were used. In addition, CPAA hydrophilicity was adjusted by conjugating zwitterionic sulfobetaine groups via tertiary amine groups, resulting in a range of different hydrophilic CPAA‐ZS. Hydrophilicity affects the hydrolysis of the polymer through fascinating water absorption. The obtained CPAA polymers showed tunable biodegradability (through zwitterionic sulfobetaine groups), low cytotoxicity, and proper wet adhesion. Wet adhesion of CPAA was higher compared to CPAE, which was correlated to the higher catechol content of CPAA. In vivo investigations on the rat model, exhibited that CPAA glue is managed to hasten the wound healing process while preventing scar formation. Accordingly, these glues can be potentially used in wound closure applications such as cosmetic surgeries.

Scar management after severe burn injuries has attracted much attention. Stem cell‐based therapies, pharmacological methods, and surgical methods have been utilized for burn scar management.^248^ Catechol chemistry can also be used to burn injuries.

### 9.7 Drug/gene/cell delivery

Controlled drug delivery systems (CDDS) have been focused on since the 1950s.^249^ More recently, CDDS based on nanomaterials has attracted many interests. The releasing may be based on diffusion‐controlled, chemically controlled, solvent‐controlled, and stimuli‐controlled delivery systems are the most important delivery mechanisms. Besides, the delivery can be externally activated/modulated for stimuli‐controlled systems. These CDDSs have been vastly applied for different types of applications, such as ocular diseases, and periodontal diseases. Light/wave, pH, redox reactions, temperature, electric/magnetic fields, and ultrasound irradiation are important stimuli used in CDDSs.^250^, ^251^

As stated above, catechol moieties depend on environmental pH. This enables pH‐responsive drug delivery system design. PD‐coated PCL nanofibers were used for pH‐triggered drug delivery systems, where positively charged molecules release faster in acidic media.^252^

Thiol–catechol chemistry has been utilized to manufacture pH‐responsive mussel‐inspired polydopamine capsules for cancer therapy. The CDDS contains an anticancer drug that releases endosomal/lysosomal pH in the cells while its release rate is very slow at physiological pH. pH‐responsive Dox‐loaded protein nanoparticles were manufactured using Iron(III)–DOPA complexes.^253^ Doxorubicin release is mainly controlled by the structural alternation of the Iron (III)–DOPA complexes caused by acidic pH. In vitro assays validated that using the Iron(III)–DOPA complexes due to the cellular uptake efficiency and excellent cytosolic release, showed wonderful cytotoxicity toward tumor cells. Mucoadhesive MI hydrogels were also used to deliver drugs for application areas like mucin and buccal.^229^, ^230^ Extended localized effects of medication, prevention of gastrointestinal drug metabolism, and high patient compliance are listed as the advantages of using buccal mucosa for drug delivery systems. Suhair Sunoqrot et al.^229^ to achieve an enhanced gastro‐retentive oral drug delivery system, designed a mussel‐inspired chemistry/methoxy poly(ethylene oxide)‐block‐poly(ε‐caprolactone) (PEO‐b‐PCL) copolymer having different mass ratios and evaluated them through in vitro tests. In another study, chitosan, as a subclass of mucoadhesive hydrogels, was functionalized using MI chemistry, then the polymer was crosslinked using genipin to be utilized as a novel drug carrier. Although catechol groups do not influence the gelation time and the mechanical properties of chitosan hydrogel, using these composite patches will not result in any inflammation. A new kind of patch for cardiac issues was introduced by Young Min Shin et al.^254^ This research group successfully used PDA‐coated cell‐adhesive peptides to functionalize a fibrous platform fabricated using the electrospinning technique. Through the in vivo tests, they applied the biomimetic fibrous cardiac patch for C2C12 myoblasts delivery. A combination of collagen/alginate core‐shell was utilized as mesenchymal stem cells (MSCs) delivery structure.^255^ This composite is supposed to perfectly encapsulate and deliver the tissue cells into damaged points including defective bone tissues.^256^

### 9.8 Antibacterial activity

**FIGURE 21:**
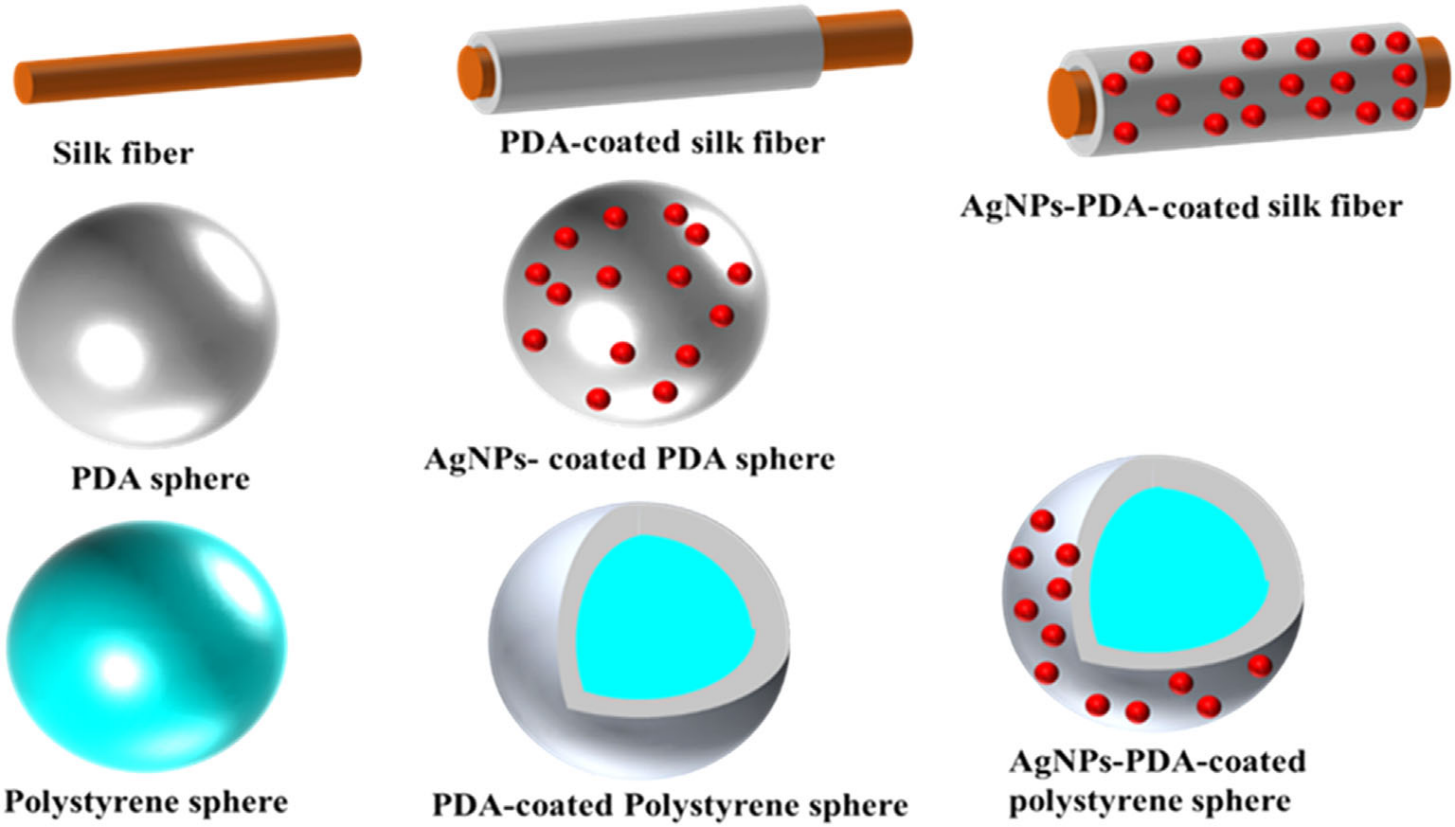
Various types of PDA‐coated materials for antimicrobial applications^146^, ^257^

A vast demand for wound healing bandages, bone replacement surgeries, and implanted medical devices have now developed into a significant, continuing issue that is sincerely concerned with the persistent existence of a vast variety of bacteria in the vicinity of these impaired locations. It is proven that the infections induced by these bacteria following surgical intervention can be fatal. Novel and useful antibacterial agents are, therefore, desperately needed to be introduced by pharmaceutical and researchers in the medical field to assure the success of implantation and organ substitution (Figure 21). For example, the orthopedic implant infectious diseases could occur mainly by staphylococcus bacteria while they colonize in the gap between tissues and the orthopedic prosthesis, causing chronic osteomyelitis and decreasing the success rate of implantation. Hence, addressing this dearth, spurred a substantial development of metallic and multimetallic antimicrobial agents (especially silver) coated with mussel‐inspired biomaterials. In general, the active catechol moieties and existing amine groups of PDA, have made it a natural, strong adhesion, and a gentle reducing agent for noble metallic cations, which is so fitted as a promising coating agent. Immobilization of tunable size silver nanoparticles onto the surface of PDA spheres, as the well‐known MI chemistry, leads to finding an excellent antibacterial material which is emerged vigorously against both classifications of Gram for bacteria.^257^

Su et al.^258^ investigated the antimicrobial activity of PDA coated onto plastic, glass, stainless steel, and gauze. These polymer coatings were manufactured by a quick shaking‐assisted approach and their bactericidal ability against two different classes of Gram‐positive/Gram‐negative bacteria (Gammaproteobacteria: *P*. *aeruginosa* and *E*. *coli*; and *Bacilli*: *S*. *aureus*). The obtained results indicate the alteration in morphology of PDA coatings with a smooth surface (sPDA) by applying a mechanical shaking technique thorough the process of self‐polymerization of dopamine in the mild basic region to roughened polydopamine (rPDA) coatings, appreciably enhanced the antimicrobial ability of it. Also, steam sterilization has been discovered ineffective in the antibacterial characteristics of the rPDA coatings. Ying Cong et al. synthesized a new antimicrobial agent using metal nanocrystals (Ag) onto polystyrene spheres, which were coated and covered by PDA. Their study showed that polystyrene‐PDA/Ag composites have a great bactericidal capability against the mostly known bacterial species.^259^ Also, many biomedical researchers tried to combine various 3D scaffolds with PDA for tissue engineering applications and mussel‐inspired hydrogels incorporation with Ag nanoparticles, which also resulted in the improvement of their antibacterial features of them.^260^ Moreover, for the garment industry and textile applications, Zhisong Lu et al.^146^ and his colleagues introduced an antibacterial material based on the synthesis of AgNPs through a redox reaction of Ag cation onto PDA‐coated silk fibers surface. Their study expects that for as‐prepared AgNPs‐PDA‐coated silk, there be a more antibacterial lasting impact than the AgNPs‐coated silk due to a greater dosage and more extended period of silver cation release demonstrating AgNPs loading in high content. In the following figure, various types of PDA‐coated materials for bactericidal uses were illustrated.

### 9.9 Therapeutic agents and cancer therapy

Application of the mussel‐inspire strategy to fabricate super materials for photothermal therapeutic (PTT) uses (e.g., cancer therapy, self‐healing hydrogels), has recently drawn increasing interest. According to the latest news of the World Cancer Report, the population who are suffering from cancer will increase to 15 million cases by 2020, so cancer therapy using photothermal methodology has become so important. Diagnostic (e.g., contrast agents) and therapeutic agents (e.g., photothermal therapy agents) coating with PDA were discussed earlier. Here, we emphasize catechol‐based chemicals which are directly used as therapeutic agents. PDA can absorb NIR radiation.

Radiotherapy and chemotherapy strategies are standard cancer treatment methods that can cause systemic cytotoxicity. However, as a minimally invasive method and with minor side effects, phototherapy (PTT) has attracted much attention.^261^ In combined photodynamic (PDT) and photothermal (PTT) therapy, hydrophilic PDA nanoparticles can be used as drug nanocarriers to kill bladder cancer cells.^262^ The therapeutic agent consists of a hydrophobic photosensitizer drug (chlorine e6, Ce6) dispersed through interaction with PDA nanoparticles. The interaction between PDA and drug is proposed as the mechanism for long‐lasting drug release. It was observed that cell uptake of PDA nanoparticles remarkably increases when Ce6 is incorporated. After irradiation with 665 nm red light, more effective therapy was observed for combined therapy. This strategy was proposed as a general method for mucosal drug delivery (such as intestine and bladder) in which much penetration of hydrophobic photosensitizer is very limited.

**FIGURE 22:**
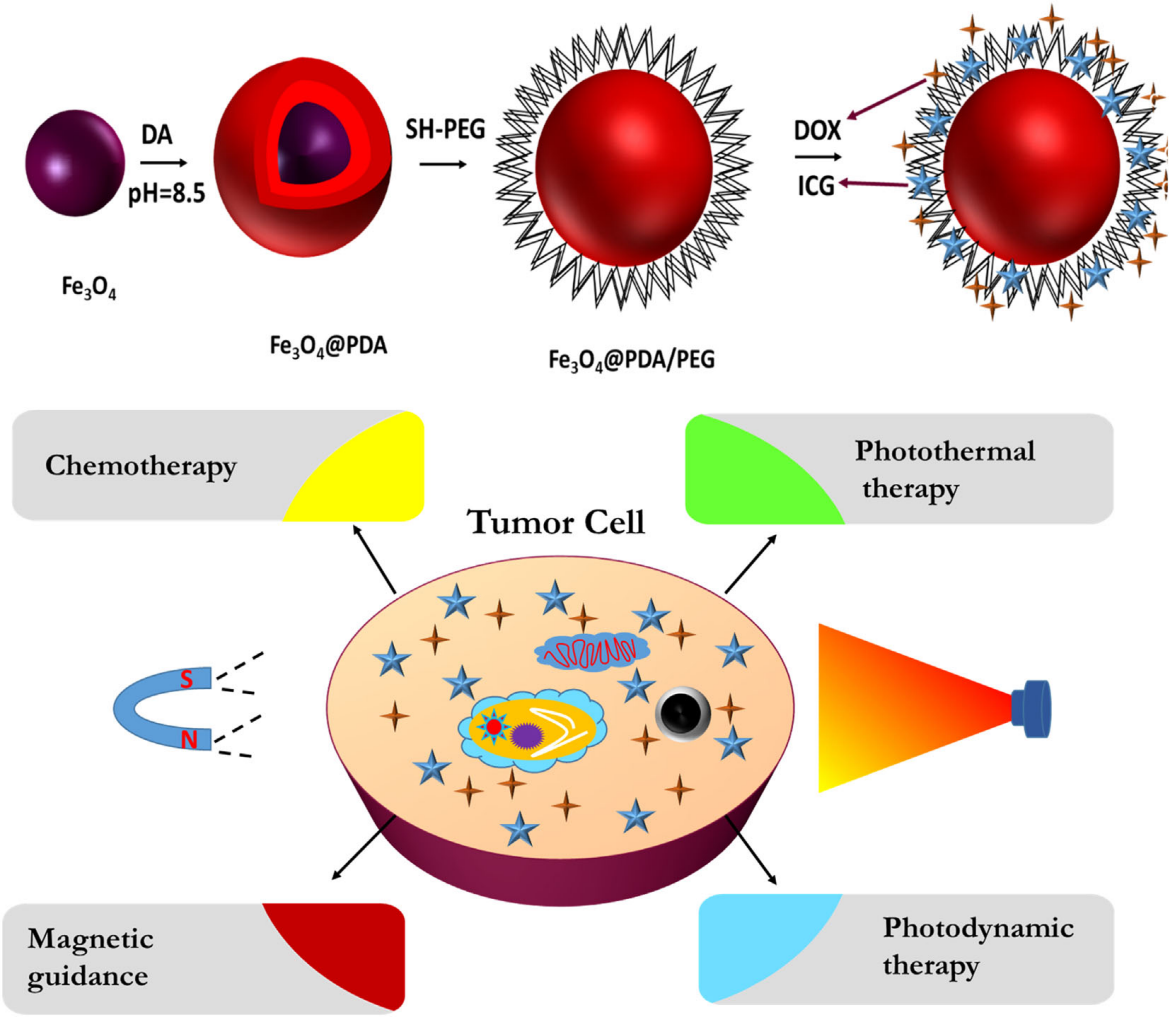
Mussel‐inspired therapeutic agents for cancer therapy^263^

Combined PDT, PTT, and chemotherapy using multifunctional therapeutic agents have also been used for cancer treatments.^263^ Magnetic Fe_3_O_4_ coated nanoparticles with a PDA‐layer and surrounding PEG corona were used for cancer treatment applications (Figure 22). In addition, indocyanine green (a diagnostic dye approved by the FDA) and doxorubicin (a naturally based anti‐cancer drug) were loaded into nanoparticles through hydrophobic interactions and π−π interactions. Under NIR irradiation, the obtained biocompatible nanocarriers produce singlet‐oxygen. Furthermore, in vitro investigations revealed that synergistic effects (such as ROS generation and DOX release) result in the effective killing of Hela cells. Accordingly, these nanosystems can act as multifunctional therapeutic agents for multimodal cancer treatments.

CD44 receptors are glycoproteins that are overexpressed by cancer cells and are believed that contribute to cancer development and progression.^264^ Hyaluronic acid is one of the significant CD44 ligands. Thus, hyaluronic acid has been vastly used in cancer therapy applications.^265^ Kim et al. fabricated carbonized fluorescent polydopamine nanoparticles (FNPs‐PDA) coated with an HA‐based shell.^266^ A prestigious study on organic PDA coated on inorganic NaYF_4_:Nd^3+^@NaLuF_4_ cores indicates that PDA shell thickness plays an important role in photothermal conversion efficiency. Unique characteristics of synthesized PDA@NaYF_4_:Nd^3+^@NaLuF_4_ such as its biocompatibility and temporal‐specific tumor imaging induced in a wonderful tumor ablation by PTT approach.^156^ Liu et al.^267^ with their colleagues, introduced a strong PTT agent consisting of PDA‐coated melanin, a nature‐inspired biopolymer, which is synthesized in a mixture of ammonia aqueous solution and ethanol. Without any side effects on healthy tissues, they provided dopamine–melanin colloidal nanospheres, which show a quick, high efficiency of tumor cells ablation even under low laser power density conditions. The synergetic effect of immobilization of polydopamine/boron nitride nanosheets (BN) onto poly‐p‐phenylene benzobisoxazole (PBO) fibers leads to the formation of a recyclable, interfacial self‐healing composite with elevated PTT properties.^268^

### 9.10 Implantable devices

Polytetrafluoroethylene (PTFE) is a well‐known, superior biomaterial, which is widely applied as a prosthesis to treat Congenital Diaphragmatic Hernia defects.^269^ Considering the advantages of covering porous expanded PTFE (ePTFE) implants in a face‐specific manner through the smart use of polydopamine, many efforts are being made to manufacture new implants featured by high cell adhesion, high biocompatibility, and excellent biodegradability for diaphragmatic replacements.^270^, ^271^ An ePTFE membrane can contain two sides with different functionalities. The side which is supposed to be in contact with the abdominal face required a weak ability of cell adhesive the same as the nature of ePTFE.

Conversely, the other side, thoracic exposed faced, should possess an excellent tissue adhesive property. So, the smart coating method is so important in this issue, and MI chemistry plays a pivotal role in this regard. Wang et al.^272^ did a large study on surface modifications of various platforms for implantation such as biopolymer, biometal, and bioceramic through bio‐interfacial functionalization of polydopamine self‐assembly process for implant uses.

Implanted devices such as electrodes could be manufactured using MI chemistry.^273^ A general scheme for biofunctionalization of the neural interface was proposed by Kang et al.^207^ The most important advantage of MI conductive hydrogels for application in implantable devices is that they can tolerate both wet conditions and temperature fluctuations.^274^

### 9.11 Electronic skin/wearable electronic

Human skin includes a network of different sensors, which obtain various environmental information and send them to the brain. The information is in particular concerned with thermal and tactile stimuli. An ambitious goal is to manufacture an electronic skin (named E‐skin) inspired by human skin, which means to develop a network of sensing elements embedded in tough, high stretchable, skin adhering, and biocompatible materials with good cell affinity. More ambitious goals are to supply these artificial skins with more functionalities such as chemical sensing, diagnostic and monitoring capabilities, odor, and taste sensing (electronic nose and tongue).^275^ More efficient robots can be designed through the application of these smart skins. Self‐healing and adhesion to human skin capabilities are essential for E‐skins. Catechol moieties, as discussed earlier, show self‐healing capabilities. High stretching also can be achieved using catechol‐containing hydrogels. These properties have made them attractive materials for developing electronic skin and wearable electronic applications. For example, a strain sensor with high sensitivity has been manufactured using polyacrylamide hydrogel and dopamine‐modified talc nanoflakes.^276^ This hydrogel shows enhanced cell affinity compared to pure PAAM.

For electronic applications such as E‐skins, electrical conductivity is an essential property.^277^ In this regard, conductive species, such as carbon nanomaterials and conducting polymers, can be included in catechol‐modified hydrogels. A sensor for monitoring human motion was designed through the incorporation of single‐wall carbon nanotubes in polyvinyl alcohol and polydopamine hydrogel.^278^ The hydrogel shows self‐healing and skin adhesiveness while it creates no cytotoxicity.

Conductive hydrogels based on dopamine‐modified reduced graphene oxide and PAAM can be used in motion monitoring and as an electromyography (EMG) electrode, as well.^273^ The hydrogel was used as an implanted EMG electrode. Furthermore, it was observed that the conductive hydrogel improves the proliferation and adhesion of the stem‐cell of the bone marrow (BMSC), which was also associated with catechol interactions with amine and thiol groups on the cell membrane and with the membrane. Electrical stimulation also affects the cell growth rate on the conductive hydrogel.^279^

### 9.12 Tissue engineering

#### 9.12.1 Tissue adhesives

**FIGURE 23:**
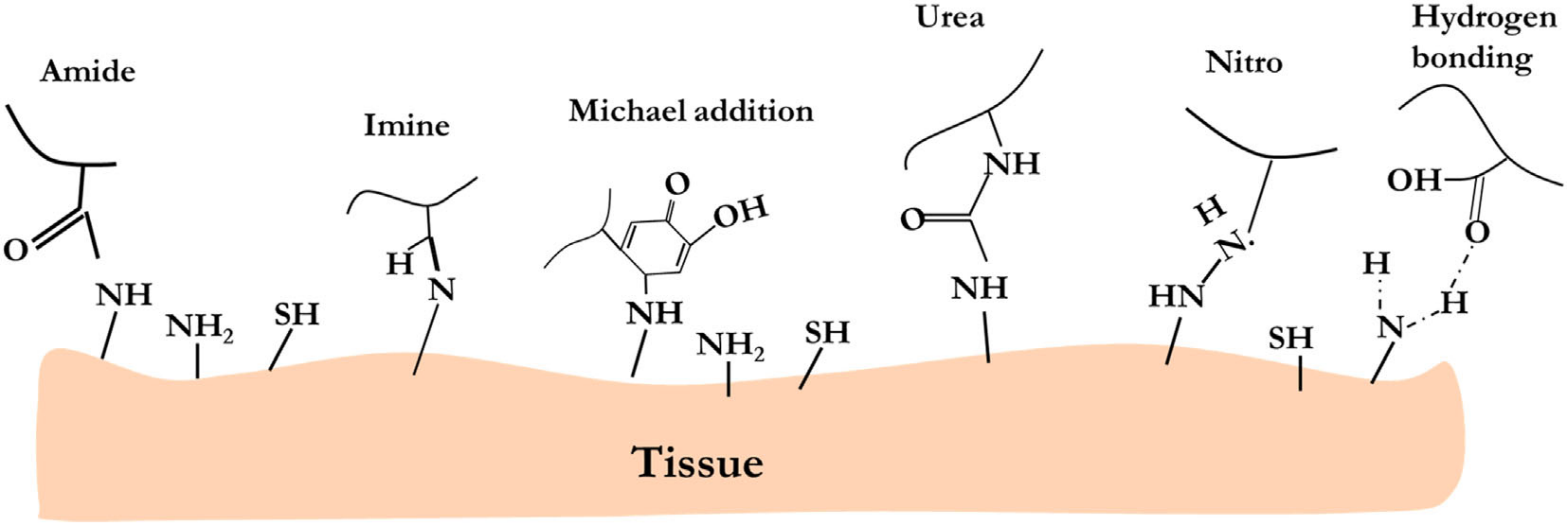
Illustration of reaction between tissue and dopamine functional groups^229^

Fibrin glue and cyanoacrylates are marketed, and conventional tissue adhesives are often used in surgery. However, poor wet adhesion, poor cytocompatibility, and higher suture costs are disadvantages that lead to new biomimetic tissue adhesives.^230^ These adhesives have been inspired by barnacles, caddis fly larvae, sandcastle worms, and mussels.^280^ Sealing the tissues, wound closure, and stopping bleeding are some applications of bioadhesives in surgery. Many mussel‐inspired bioadhesives have been designed using MI chemistry. Figure 23 shows possible reactions between the tissue surface and dopamine.^229^

**FIGURE 24:**
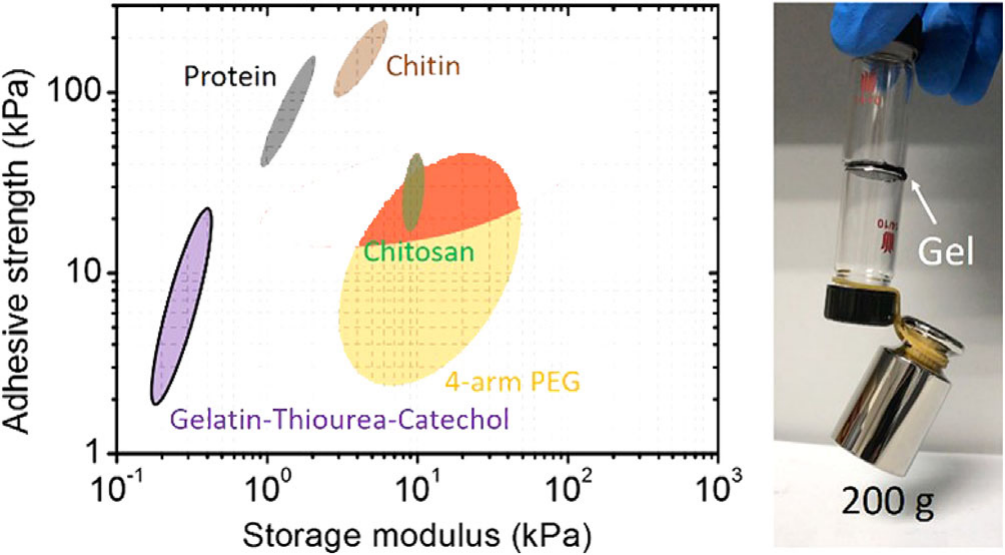
Adhesive strength versus storage modulus for different biomaterials^284^

The adhesive strength of the MAP is 400–1000 kPa, considerably better than commercial fibrin glue (~15 kPa).^281^, ^282^, ^283^ Most commercial glues are concerned with developing bioadhesives by imparting catechol‐containing moieties into biocompatible polymers, usually as side chains. Figure 24 presents the adhesive strength of diversified biopolymers versus storage modulus. Polysaccharide‐based mussel‐inspired bioadhesives, including dextran, alginate, and chitosan, have been manufactured through catechol functionalization.^284^

#### 9.12.2 Bone, cartilage, and dental replacement materials

Bone is the most rigid body part found in vertebrates, responsible for supporting and protecting various body organs and giving us the ability for numerous motions. It produces red and white blood cells and stores minerals. Bone tissue, also known as osseous tissue, is composed of cells (e.g., osteocytes, osteoblasts, and osteoclasts), fibers (e.g., type I collagen), ECM, and embedded blood vessels and nerve fibers. The ECM of bone tissue is composed of organic materials (e.g., collagen fibers which make up 90% of the organic bone fraction, proteins, and polysaccharides) and minerals (e.g., hydroxyapatite which accounts for 50–70 wt% of bone,^285^ calcium carbonate, and various ions such as magnesium, sodium, sulfate, and fluoride).^286^ Collagen fiber supplies the osseous tissue with tensile strength while small platelet‐like hydroxyapatite crystals (Ca_10_(PO_4_)_6_(OH)_2_) endow the compressive strength, while synergistic effects are also observed. A biomimetic approach has resulted in the design of scaffolds composed of hydroxyapatite and collagen.^287^ Adhesion of osteoblast onto the implant's surface determines the implant's biocompatibility.^288^ Surface modification was considered a key strategy to increase the aptness of bone implants in orthopedics and dentistry.^289^ Also, implant surface modification using calcium phosphate derivatives such as hydroxyapatite has been widely used in bone implants and bone regeneration.^290^, ^291^ Hybrid composites were fabricated successfully with desired characteristics to optimize their condition for bio application goals, including bone tissue engineering purposes. The test results exhibited that hydroxyapatite tended to grow faster in porous structures like zeolite crystals than in pure polymeric scaffolds with low porosity like chitosan where few hydroxyapatites were detected. Microporous surface assisted hydroxyapatite cells to grow more extensively and shape hydroxyapatite layers on targeted bone cultures. Also, calcium cations in crystal structures of zeolites elevated the process of hydroxyapatite mineralization in comparison with the samples without any calcium in their framework.

Bone‐like apatite layers in tissue–implant interfaces can enhance biocompatibility. Simulated body fluid (SBF) immersion is a biomimetic process widely used in the creation of apatite‐based coatings.^292^ However, it has recently been reported that the formation kinetics of amorphous calcium phosphate (ACP) (raw material for bone‐like apatite) deposition on tricalcium silicate (TCS) can be improved through MI chemistry.^212^ In this regard, they modified the TCS using a polydopamine coating. SBF immersion results showed a nearly two‐fold enhancement in the thickness of bone‐like apatite for PDA‐modified TCS relative to bare TCS. They pointed out that the abundance of calcium ions and decreased interface energy of ACP and PDA‐coated TCS are contributing mechanisms for this phenomenon. Wei et al. also investigated the exceptionally high capacity of co‐acerbated Mfp‐3S for hydroxyapatite.^17^

Many researchers used different nanomaterials to mimic bone tissue's high mechanical properties.^293^, ^294^, ^295^ Graphene and its derivatives gained much interest in bone tissue engineering due to their unique properties and structure.^296^, ^297^ GO's biocompatibility allows scaffold adhesion, proliferation, and differentiation.^296^, ^298^ Accordingly, prepared composites exhibited high mechanical strength, too. This is mostly attributed to the extremely strong graphene oxide bonds that endured high amounts of tension.^297^

On the other hand, GO coatings can modify the bio‐inert nature of Titanium (Ti) scaffolds to increase their insufficient osteoinductivity and enable it to integrate with the bone tissue. However, due to the inert chemical surface of Ti, the uniform deposition of GO onto the Ti is very hard. Nevertheless, MI chemistry is a robust strategy for surface modification of versatile surfaces, including inert metals. Accordingly, Han et al.^50^ coated the porous Ti scaffolds using polydopamine, followed by a coating of GO nanosheets. Graphene oxide nanosheets and other materials like that such as zeolites^299^ and gelatin polymer blends which have proved to be applicable composites in different internal tissue media^300^ coatings make them perfect choices for toxic substances and ion removal from alloy implants area. Combining titanium within the high surface area and porous framework of zeolites provides perfect adhesion to the substrates by replacing the titanium with its rather hydrophobic surface with hydrophilic interconnected pores that can inhibit implant loosening, which is considered a very satisfying quality for bio‐implants. These coatings, in addition to titanium implants, decrease the modulus mismatch with bone tissue and improve implant osseointegration.

Chen et al.^301^ manufactured a 3D‐printed scaffold with poly(caprolactone) (PCL) loaded with polydopamine‐modified calcium silicate (PDACS) and Wharton's jelly mesenchymal stem cells (WJMSCs). The scaffold's pores were filled with a hydrogel of alginate and gelatin containing human umbilical vein endothelial cells (HUVEC), which shows improved mechanical properties and tissue adhesion. Furthermore, cell incorporation has resulted in enhanced osteogenesis and angiogenesis as well. Observation showed oxidative cross‐linking of dopamine coated on alginate 3D scaffold decreased cytotoxicity and improved the preservation of different stem cells in the gel framework for osteogenesis tissue regeneration.

Gelatin‐based hydrogels methacryloyl attracted considerable attention in cartilage regenerative engineering due to high biocompatibility. However, these hydrogels' mechanical properties do not meet the requirements so modifications are necessary to increase mechanical properties. Gan et al.^302^ modified gelatin methacryloyl using MI chemistry for regenerating the cartilage. The intercalated dopamine methacrylamide (DMA) oligomers as a polymerizable precursor into the GelMA network to enhance the final hydrogel's mechanical properties. DMA oligomers interact with each other through different physical interactions while bonding to GelMA chains covalently. Consequently, robust and resilient hydrogels with excellent stability are created. Besides, enhanced cell affinity and improved tissue adhesion were observed.

Glycosaminoglycan is a significant component of cartilage ECM. Chondroitin sulfate is sulfated glycosaminoglycan used in osteoarthritis.^303^, ^304^ However, hydrogels which are based on glycosaminoglycan repel cells due to negative surface charges. Han et al. made a tissue‐adhesive hydrogel with polydopamine, chondroitin sulfate, and polyacrylamide for growth‐free cartilage TE.^305^ While polydopamine improves cell affinity and tissue adhesiveness, chondroitin sulfate promotes chondrogenesis.

Wang et al. also researched bioactive peptide application to alter bone tissue engineering materials,^306^ where they investigated different routes of synthesis for modification of bone tissue repairs. Such mechanisms include electrodeposition, covalent immobilization, physical adsorption, and other modern literature methods. Different types of peptides were suggested and examined as solid nominees for the bone repair treatment, including ECM‐derived tissues, BMP‐derived ones, and some other limited‐use samples. These peptides can trigger some unique signaling points that regulate osteogenic‐related cellular interactions.

Dental applications of new series of tissue technologies have recently gathered much attention. For example, Lee et al.^307^ fabricated catechol‐functionalized synthetic polymers for composite restoration purposes emplaced for playing the role of dental adhesive to contaminated dentin culture. The study showed that the extending of polymer chains by this approach could as well ease physical crosslinking for a variety of polymer chains by entanglement. Furthermore, the catechol moieties undergo chemical coupling reactions leading to crosslinking, although there is no definitive reason to suggest the involvement of catechols in this adhesive. These impacts will enhance the polymer film layer's mechanical performance, which elevates cohesive bonding. The improved interfacial and cohesive bonding enhanced the shear strength when the solvent was removed from the adhesive after drying than in the wet condition. From another point of view, adding water to the control adhesive sample diluted the monomer concentration, which could have induced incomplete polymerization, thus decreasing the bond's mechanical strength.^307^, ^308^

To increase hydroxyapatite formation, scientists also recommended a template‐free electrochemical polymerization method, which founded to be efficient in implants' success. For instance, Wang et al.^309^ followed this synthesis route to fabricate catechol‐embedded electroactive polypyrrole nanowires on the interface section of titanium implants to enhance hydroxyapatite formation. Adhesion degree is a crucial feature inside the human body for the bioactive layer‐coated metallic implants. Results of the study revealed that only 15 min of ultra‐sonication does not kill composite nanowire coatings. The CaP agglomerations were created on the PPy/PDA nanograins' surface after 3 days of incubation, and the agglomerates sheltered most of the PPy/PDA nanowire composite surface within 3 days, mostly associated with the free PPy/PDA nanowires concentration of catechols much larger than the calculated PPy/PDA nanograins. The number of agglomerates on the surface of PPy/NSA nanowires was much lower than on PPy/PDA nanograins and nanowires, showing that PDA catechols are important for the CaP nucleation process. A week after incubation, CaP minerals smoothly covered the titanium substrate enhanced by the PPy/PDA nanowires, improving the formation of hydroxyapatite by improving morphological characteristics. The high surface area of the microporous structure seems to be a key point of cell proliferation and its uniform roughness, and the semi‐hydrophilic surface increases the adhesion amounts between them.

#### 9.12.3 Vascular system

There are increasing demands for vascular scaffolds for clinical applications. Electrospun scaffolds with high mechanical strength and flexibility have gained much attention. Lee et al.^310^ fabricated a three‐dimensional bi‐layered scaffold using PCL and manufactured vascular grafts with porous structure. Immobilizing vascular endothelial growth factor (VEGF) on PDA‐modified tubular scaffolds also increased vascular cell proliferation and enhanced angiogenesis. Biotin‐induced immobilized VEGF labeling revealed that fluorescence peaks increased as a function of VEGF solution content. VEGF's effects on HUVEC adhesion were inconsiderable, which should have been protected by creating polydopamine film, which also supported cell adhesion.

**FIGURE 25:**
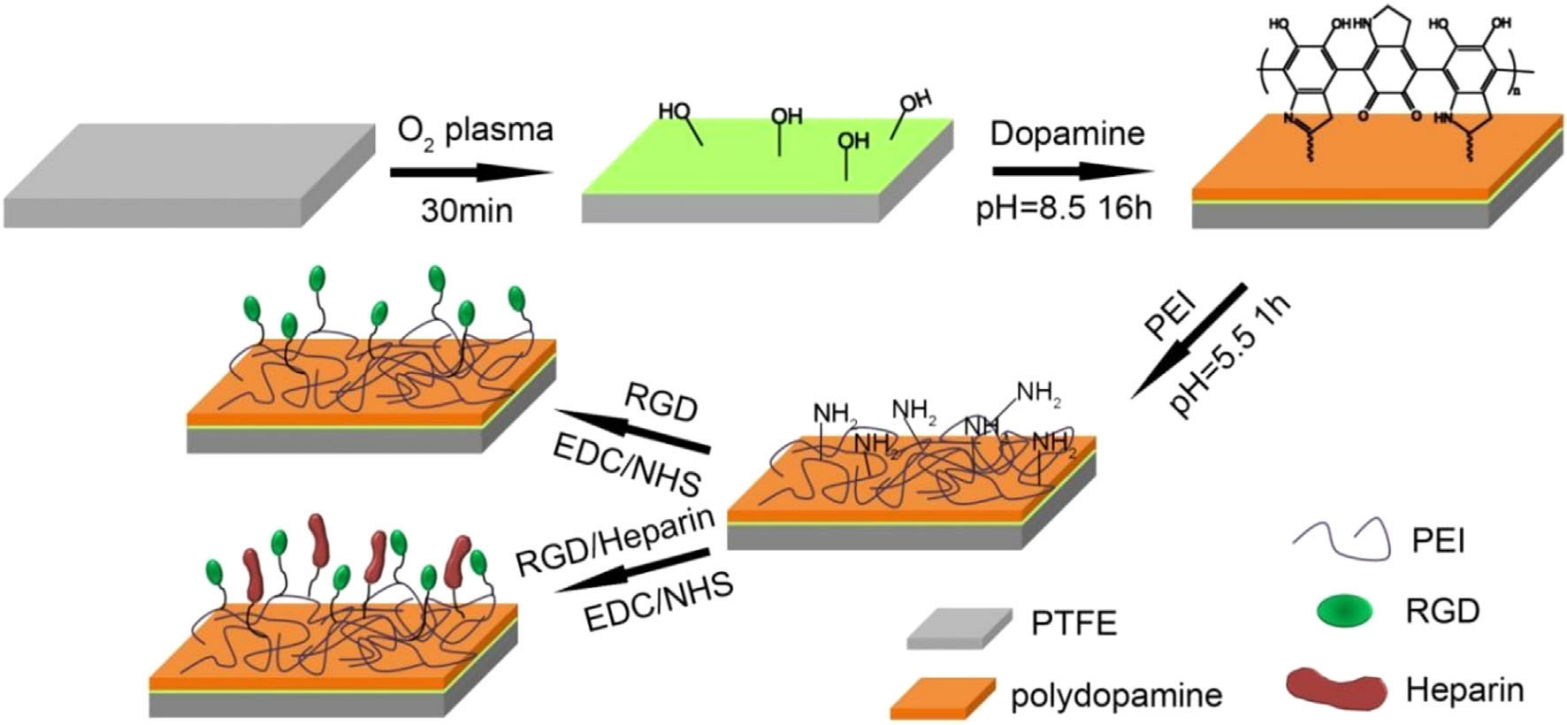
Schematic immobilization of RGD/heparin on dopamine/polytetrafluoroethylene film^309^

Coating polydopamine on the surface of PLCL improved its tissue adhesion and simplified the immobilization of dual factors (RGD peptide and bFGF), which can be used as endothelial vascular graft components.^311^ Likewise, after oxygen plasma treatment of polytetrafluoroethylene surface to increase its hydrophilicity, dopamine was coated on the surface to facilitate immobilization of RGD and heparin. The resulting increment of platelet adhesion to the complex was remarkable after dopamine coating and RGD immobilization (Figure 25).^215^

#### 9.12.4 Neural system

The central m (CNS) and peripheral nervous systems (PNS) create our entire nervous network. Traumatic injuries may result in damage to CNS or PNS. While CNS possesses a minimal regeneration capability, PNS can regenerate itself to some extent.^312^ Improving the regeneration process in PNS has triggered numerous research works resulting in various therapeutic strategies such as grafting autologous and nerve guidance conduits (NGCs). NGCs make a bridge between nerve defects and support adequate growth of axons. Chen et al. manufactured a 3D‐printed nerve conduit using PCL coated by a decellularized extracellular matrix (dECM) and PDA.^313^ dECM, which is a biomaterial secreted by cells, has been recently focused on biomedical applications.^314^, ^315^ The PDA coatings facilitate the immobilization of dECM onto the conduit.

On the other hand, electrical stimulation can improve the growth rate of axons and neurite differentiation such that conductive materials (e.g., graphene and CNT) have been used for manufacturing scaffolds for nerve regeneration. PCL loaded with 3D porous graphene (SG) or multi‐layer graphene (MG) scaffolds were fabricated using a 3D printer and layer‐by‐layer casting (LBLC) techniques.^316^ PCL scaffolds were treated with PDA and arginyl glycyl aspartic acid (RGD) to facilitate cell binding. In vitro and in vivo studies showed remarkable neural expression enhancement, axon production, and demyelination.

#### 9.12.5 Skeletal muscle

Skeletal muscle endogenous progenitor cells, that is, satellite cells, show a substantial degree of proneness to muscle differentiation, showing similar characteristics and associations to the muscle of the parent. This indicates that using a suitable progenitor cell, specifically in facial muscles, tissue engineering seems to be crucial, which possesses a unique anatomical and fiber blend, if it is analyzed according to other skeletal muscles. Muscle tissue engineering needs a well‐structured scaffold for its configuration support and modulates the proliferation and differentiation of muscle progenitor cells.

In a research conductive nanofibers in which their architectures were manually well balanced were studied for their use in skeletal muscle tissue engineering.^317^ Polyaniline (PANi) and poly‐π‐caprolactone (PCL) composites were synthesized by electrospinning in presence of a directed magnetic field. The outcome suggested that this kind of tissue sample has the capability of ordering myoblast orientation and improving myotube generation.

In another research, surface improvement and topographical signals of mussel‐inspired samples were demonstrated to understand their influence on skeletal myoblasts manners.^318^ Cell proliferation tests have been performed on PDA‐modified samples by MTT assay. For the PDA‐modified condition, the absorbance measured at 595 nm, used for measuring cell density, increased by almost 24% within 2 days and by 50% within 4 days in comparison with results obtained for unmodified glass. This indicates further improved myoblast proliferation by the PDA modification of the glass substrate. The physical parameters of the PDA layer, like its thickness, surface roughness, and surface coverage, maybe differed by altering the rates of dopamine concentration and its solution immersing time. The significant improvement of myoblast proliferation is associated with the rectification of surface chemistry after PDA modification.

#### 9.12.6 Ocular tissue

Dopamine can be found in human aqueous humor, trabecular meshwork, tear, iris, uveo‐sclera, intermediate as well as peripheral parts of the cornea, and plasma. Dopamine participates in the cascade that regulates visual development. It is released due to retinal responding to light, which has a pivotal role in light adaption and induced myopia. Interaction of dopamine with myopia restricts myopia growth.

Drug‐resistant saprophytic fungal pathogens and bacteria have caused severe pain, lasting cell damage, infections, and ulceration, which can lead to loss of vision and various other ocular diseases. Newly designed contact lenses, not only can sustain and targeted release of the drugs, more effective curative efficiency at a longer rate than eye drops but also cause smaller side effects.

Due to the unique characteristics of dopamine, such as the functionalization ability, wet adhesion, antimicrobial properties, noninflammatory properties, and high visible light transmittance, MI‐compounds are applied widely as a suitable coating for ocular disease treatments. An in vivo analysis of the multilayer development of silver nanoparticles on the contact lens surface coated by dopamine found that due to the higher release rate of silver cations from the dopamine layer, the antibacterial behavior of the lens and the auto‐oxidation of dopamine led to a higher visible light transmission of the lens.^319^ Optimizing the silver particle size and concentration, which is covered by the optimal amount of dopamine, exhibits a practical curative impact on bacterial and fungal keratitis hybrid treatments.^319^

## 10 CLINICAL APPLICATIONS

Although mussel‐inspired biomaterials have vastly been studied and exploited on the lab scale, still several practical challenges are remained unsolved to improve the translation of these compounds for human trials. Noteworthily, one of the most important issues is to protect catechol moieties from unwanted oxidation. Moreover, the biodegradability of PDA is another factor that should be considered to ensure the success of dopamine‐conjugated biomaterials for clinical trials.^320^, ^321^

Nevertheless, a few numbers of clinical trials on catecholamine chitosan‐based hemostatic pads have been undertaken. For instance, InnoSEAL Plus which is introduced by InnoTherapy in 2015 is a coagulation factor‐free catecholamine chitosan‐based bioadhesive to control intraoperative/postoperative bleeding. The proposed mechanism for this inexpensive product is based on a quick reaction with plasma protein in the blood and simultaneously production of the hemostatic pellicle. This mechanism is independent of the human blood coagulation system and thereby is expected to excel other fibrin‐based sealants when it comes to dealing with patients with the disorder in normal blood coagulation. To date, this FDA‐approved pad has been subjected to different single and multi‐center open‐label randomized controlled clinical trials in high‐income economy countries such as South Korea, as well as lower‐middle‐income countries such as Pakistan.^322^, ^323^ In 2020, Aijaz et al.^323^ studied the efficacy of InnoSEAL pad in a combination with the transracial band (TRB) on 714 randomly selected adult patients to enhance hemorrhage and better control bleeding. Their findings justify a significant improvement of this combinative method in terms of ease of use, short hemostasis time, and less discomfort for patients. The following figure demonstrates photography images of the InnoSEAL pad applied in human trials at Tabba Heart Institute (THI) Karachi, Pakistan.^322^ Very recently in 2021, Choi and coworkers^322^ have been reported a multicenter single‐blinded clinical study on the efficacy of InnoSEAL Plus to stop postoperative bleeding in 96 hepatectomy patients. This nontoxic bioadhesive showed comparable results in controlling the oozing hemorrhage from the transected liver surface only in 3 min compared to fibrin‐based TachoSil sealant. Furthermore, the one‐month follow‐up study on the applicants confirms the high efficacy success rate of InnoSEAL pads with no reported rebleeding or any other adverse events.

Expectantly, ever‐increasing in research efforts on mussel‐inspired materials in all fields of biomedical engineering from drug delivery and tissue engineering to cancer research has increased the hope for more patents and FDA‐approved products for clinical trials and real‐life applications in near future.

## 11 SUMMARY

Understanding MI chemistry as a nature‐selected mechanism has opened a new window with limitless applications for broad‐ranging of requests from environmental to medicine. From the biomedical application perspective, to develop advanced materials such as wearable devices, drug delivery systems, and surgical glues with strong adhesion to wet surfaces (e.g., chronic wounded tissues) while still keeping high mechanical properties, there is an essential need to acquire profound knowledge about MI chemistry and comprehensively study of distinctive adhesiveness of the mussel foot proteins. In this review article, we summarized and thoroughly discussed different aspects of mussel chemistry and the sophisticated mechanism of wet adhesion which could provide an excellent guideline for the design of future functional materials for diversified biomedical applications.

## AUTHOR CONTRIBUTIONS

**Ali Taghizadeh:** Investigation (equal); resources (equal); writing – original draft (supporting). **Mohsen Taghizadeh:** Investigation (equal); methodology (supporting); resources (supporting); visualization (equal); writing – original draft (equal). **Mohsen Khodadadi Yazdi:** Investigation (equal); methodology (supporting); writing – original draft (supporting). **Payam Zarrintaj:** Investigation (supporting); methodology (supporting); software (equal); validation (equal); writing – original draft (equal). **Joshua D. Ramsey:** Investigation (supporting); methodology (supporting); writing – review and editing (supporting). **Farzad Seidi:** Conceptualization (equal); investigation (supporting); supervision (supporting); writing – original draft (equal); writing – review and editing (supporting). **Florian J. Stadler:** Investigation (supporting); methodology (supporting); validation (supporting); writing – original draft (supporting). **Haeshin Lee:** Investigation (supporting); software (supporting); supervision (supporting); writing – original draft (supporting); writing – review and editing (supporting). **Mohammad Reza Saeb:** Investigation (supporting); methodology (supporting); supervision (lead); writing – review and editing (supporting). **Masoud Mozafari:** Methodology (lead); project administration (lead); supervision (lead); writing – review and editing (lead).

## CONFLICT OF INTEREST

There is no conflict of interest to declare.

## Data Availability

Data sharing is not applicable to this article as no datasets were generated or analyzed during the current study.
